# Epigenetic Gene-Regulatory Loci in Alu Elements Associated with Autism Susceptibility in the Prefrontal Cortex of ASD

**DOI:** 10.3390/ijms24087518

**Published:** 2023-04-19

**Authors:** Thanit Saeliw, Songphon Kanlayaprasit, Surangrat Thongkorn, Kwanjira Songsritaya, Bumpenporn Sanannam, Chanachai Sae-Lee, Depicha Jindatip, Valerie W. Hu, Tewarit Sarachana

**Affiliations:** 1 The Ph.D. Program in Clinical Biochemistry and Molecular Medicine, Department of Clinical Chemistry, Faculty of Allied Health Sciences, Chulalongkorn University, Bangkok 10330, Thailand; 2 Systems Neuroscience of Autism and Psychiatric Disorders (SYNAPS) Research Unit, Department of Clinical Chemistry, Faculty of Allied Health Sciences, Chulalongkorn University, Bangkok 10330, Thailand; 3 Department of Biotechnology and Biomedicine (DTU Bioengineering), Technical University of Denmark, 2800 Kongens Lyngby, Denmark; 4 The M.Sc. Program in Clinical Biochemistry and Molecular Medicine, Department of Clinical Chemistry, Faculty of Allied Health Sciences, Chulalongkorn University, Bangkok 10330, Thailand; 5 Division of Anatomy, Department of Preclinical Science, Faculty of Medicine, Thammasat University, Pathumthani 12120, Thailand; 6 Research Division, Faculty of Medicine Siriraj Hospital, Mahidol University, Bangkok 10700, Thailand; 7 Department of Clinical Pathology, Faculty of Medicine Siriraj Hospital, Mahidol University, Bangkok 10700, Thailand; 8 Department of Anatomy, Faculty of Medicine, Chulalongkorn University, Bangkok 10330, Thailand; 9 Department of Biochemistry and Molecular Medicine, School of Medicine and Health Sciences, The George Washington University, Washington, DC 20052, USA

**Keywords:** Alu elements, autism spectrum disorder, DNA methylation, epigenetic mechanism, retrotransposons, transposable elements

## Abstract

Alu elements are transposable elements that can influence gene regulation through several mechanisms; nevertheless, it remains unclear whether dysregulation of Alu elements contributes to the neuropathology of autism spectrum disorder (ASD). In this study, we characterized transposable element expression profiles and their sequence characteristics in the prefrontal cortex tissues of ASD and unaffected individuals using RNA-sequencing data. Our results showed that most of the differentially expressed transposable elements belong to the Alu family, with 659 loci of Alu elements corresponding to 456 differentially expressed genes in the prefrontal cortex of ASD individuals. We predicted cis- and trans-regulation of Alu elements to host/distant genes by conducting correlation analyses. The expression level of Alu elements correlated significantly with 133 host genes (cis-regulation, adjusted *p* < 0.05) associated with ASD as well as the cell survival and cell death of neuronal cells. Transcription factor binding sites in the promoter regions of differentially expressed Alu elements are conserved and associated with autism candidate genes, including *RORA*. COBRA analyses of postmortem brain tissues showed significant hypomethylation in global methylation analyses of Alu elements in ASD subphenotypes as well as DNA methylation of Alu elements located near the *RNF-135* gene (*p* < 0.05). In addition, we found that neuronal cell density, which was significantly increased (*p* = 0.042), correlated with the expression of genes associated with Alu elements in the prefrontal cortex of ASD. Finally, we determined a relationship between these findings and the ASD severity (i.e., ADI-R scores) of individuals with ASD. Our findings provide a better understanding of the impact of Alu elements on gene regulation and molecular neuropathology in the brain tissues of ASD individuals, which deserves further investigation.

## 1. Introduction

Autism spectrum disorder (ASD) is a neurodevelopmental disorder characterized by behavioral deficits based on the Diagnostic and Statistical Manual of Mental Disorders, Fifth Edition (DSM-5) criteria, including deficits in social interactions and communication, restricted interests, and repetitive behaviors [1]. According to the Centers for Disease Control and Prevention (CDC), the prevalence of ASD increased dramatically in 2020, reaching approximately 1 in 36 children in the United States [2]. ASD is a complex condition, even though its root causes are still unknown. Despite substantial genetic evidence over the past two decades, only 10–20% of ASD cases involve an identified genetic cause, and more than half of ASD cases are idiopathic [3]. A combination of both genetic and environmental factors influences the heterogeneity of ASD’s clinical phenotypes and severity [4,5,6,7,8,9,10,11], suggesting that epigenetic mechanisms may be the link between these two factors. DNA methylation is a key epigenetic process that contributes to the etiology of neurodevelopmental disorders, including ASD [12].

Several studies have demonstrated that alterations in DNA methylation at transcription start sites (TSSs) in ASD brains compared to control brains result in the altered expression of many genes, including *OXTR*, *SNRPN*, *MAGEL2*, *RELN*, and *GAD1* [12]. However, epigenetic changes in a single gene may not occur frequently, and genome-wide DNA methylation profiling in ASD is considered an alternative tool used for identifying differentially methylated loci contributing to ASD susceptibility. Genome-wide transcriptome and DNA methylome analyses in postmortem ASD brain tissue have revealed that differentially methylated and expressed genes are involved in synaptic development and immune function [13,14,15,16]. Nonetheless, most DNA methylation and transcriptome analyses of postmortem ASD brain tissues have focused on protein-coding regions rather than noncoding regions, including the transposable elements that account for most of the CpG sites in the human genome.

**Figure 1:**
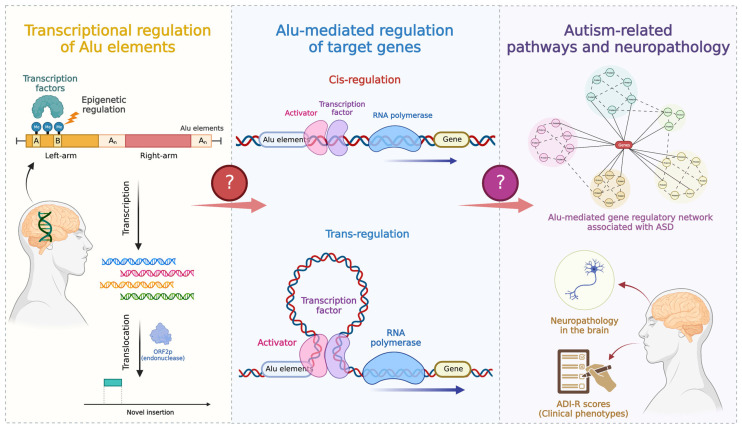
The conceptual framework of this study. This figure shows the structure and transcriptional regulation or retrotransposition of Alu elements through epigenetic mechanisms. Alu elements can act as enhancers or alternative promoters for host/target genes, resulting in transcriptome alteration because they contain several transcription factor binding sites [17,26,27]. It is unclear whether Alu elements are associated with the transcriptome, neuropathology, and clinical phenotype (ADI-R scores) of the brain tissues of ASD individuals. This figure was created with BioRender.com (accessed on 19 March 2023).

Transposable elements (TEs) are mobile genetic elements that copy and paste themselves into a new genomic location. Alu elements and long interspersed nuclear element-1 (LINE-1) comprise the majority of TEs that remain active in the human genome, comprising more than 30% of the genome at a copy number of over 1 million elements [17]. By introducing alternative promoters or enhancers, novel splicing sites, and epigenetic alterations, these elements affect the expression of host or nearby protein-coding genes [17]. Alu elements, which are 300 bp in length, are the most common short interspersed nuclear element (SINE). Alu has a dimeric structure that is derived from the *7SL RNA* gene, with each monomer linked by an A-rich region [18]. The left monomer of Alu contains the A- and B-box promoters for RNA polymerase III promoters. The right monomer of Alu elements has a poly(A) tail that is important for their retrotransposition mechanism [19]. Alu is a nonautonomous retrotransposon. The retrotransposition mechanism of Alu is similar to that of LINE-1 retrotransposition but differs in that Alu is unable to produce ORF1p and ORF2p proteins. Alu RNA requires the ORFp protein produced from LINE-1 for transport to the nucleus and target-site-primed reverse transcription [17,18,19]. At present, understanding of the precise role of LINE-1 and Alu elements in ASD development remains limited. In previous studies, we reported alterations in the global DNA methylation of LINE-1 and Alu elements in lymphoblastoid cell lines (LCLs) of an ASD subgroup based on clinical phenotype (ADI-R scores). Alterations in LINE-1 methylation were specifically observed in the ASD subtype with severe language impairment, whereas an alteration in Alu methylation was specific to ASD with mild symptoms. Moreover, several genes with LINE-1 and Alu insertions were differentially expressed in the peripheral blood and LCLs of ASD. These genes are known to be associated with neurodevelopmental disorders [20,21]. Locus- and family-specific Alu and LINE-1 methylation and the expression level of their host genes were found to be altered in the blood of ASD individuals identified using the Infinium 450 K platform [22]. Global LINE-1 methylation was significantly decreased in the blood samples from ASD individuals with mental regression [23]. In addition, whole-genome sequencing analysis of ASD brain tissues shows a higher number of novel insertions of LINE-1 and Alu elements than in normal brain tissues [24]. As reported by Shpyleva et al., (2018), binding of the MeCP2 repressor to the LINE-1 promoter was significantly decreased and correlated with an overexpression of LINE-1 in ASD brains [25]. These findings suggest that the epigenetic mechanism of LINE-1 and Alu elements may be altered in ASD brain tissues. However, it remains unclear whether Alu elements contribute to changes in gene expression levels in ASD brain tissues and are linked to the neuropathology and clinical phenotypes of individuals with ASD (Figure 1). Previous studies have focused on global DNA methylation and the expression of LINE-1 and Alu elements in the LCLs and blood of ASD individuals, but the study of LINE-1 in ASD brain tissues was limited because only total LINE-1 methylation and transcripts were determined. This is the first study to investigate the expression profiles of TEs in relation to the epigenetic regulation of TEs in the brains of ASD individuals.

In this study, we aimed to investigate relationships between TEs, the transcriptome, neuropathology, and clinical phenotype (ADI-R scores) in the brain tissues of individuals with ASD. We first collected RNA-seq datasets from postmortem brain tissues of individuals with ASD and those who were not affected from the NCBI Gene Expression Omnibus (GEO) DataSets and the NCBI Sequence Read Archive (SRA) repository to explore the expression profile of TEs that suggest TE dysregulation in ASD. The datasets were then reanalyzed for gene and TE profiling and the bedtools package was used to identify host/target genes among the intersected genomic loci between differentially expressed TEs and differentially expressed genes. To determine whether TEs can influence host/target gene expression, we performed correlation analysis between the expression value of each TE and genes by classifying TEs into those residing within exons, introns, and those found upstream from the TSS of genes. Next, to understand whether TEs are associated with ASD, we used Ingenuity Pathway Analysis (IPA) software to predict the biological functions and gene regulatory networks of host/target genes associated with TEs. Moreover, we analyzed in more detail the conserved regions and upstream regulators of dysregulated TEs in ASD. Due to the abundance of TEs in the genome, we collected DNA sequences of differentially expressed TEs (DETEs) in ASD brain tissues and compared them with sequences of nondifferentially expressed TEs using multiple sequence alignment analysis. The conserved regions were then employed to predict transcription factor binding sites (TFBSs) using the JASPAR CORE database. In addition, we conducted a pilot study using postmortem human brain tissues of ASD and unaffected individuals obtained from the Harvard Brain Tissue Resource Center and confirmed the expression levels of host/target genes selected from RNA-seq analysis. To determine DNA methylation of Alu elements, we applied combined bisulfite restriction analysis (COBRA) for global Alu methylation and locus-specific methylation of Alu located near host genes. Finally, Nissl staining was performed to evaluate the neuropathology in paraffin-embedded postmortem brain tissues. To determine the relationship between TEs, neuropathology, and ASD severity (ADI-R scores), we performed correlation analysis between the results. We expect that the results of our study will provide a better understanding of the relationship between TEs and the molecular and neuropathology of ASD.

## 2. Results

### 2.1. Transposable Element Profiling in the Prefrontal Cortex of ASD

**Figure 2:**
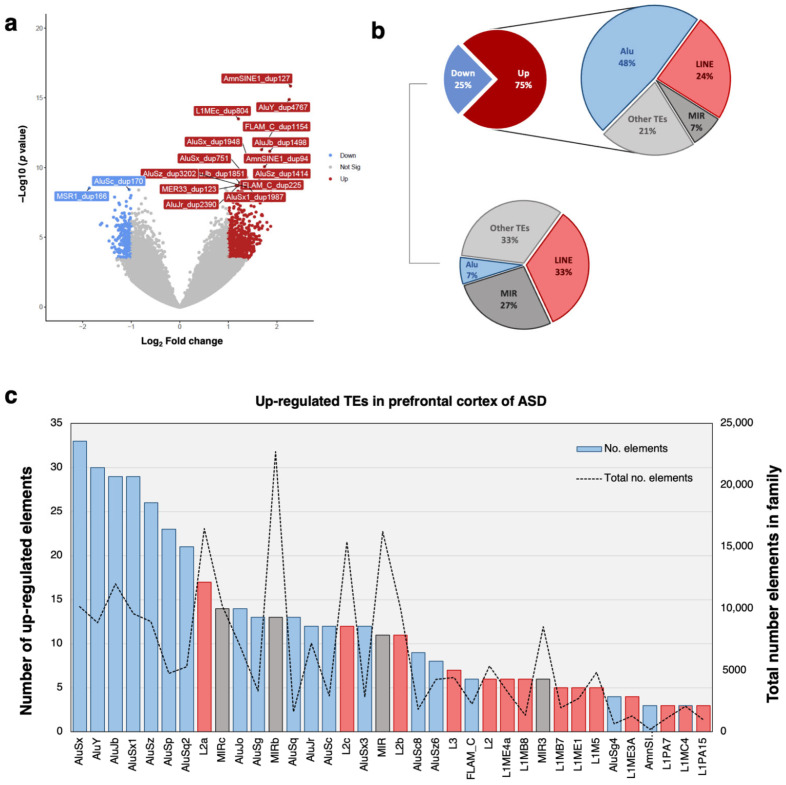
Transposable element profiling in the prefrontal cortex of ASD individuals: (**a**) Volcano plot of all individual TEs in the human genome. Dysregulated elements are labeled at a significance threshold (BH-adjusted *p* < 0.01 and log_2_FC > 1), and the most significant elements are labeled with a symbol. (**b**) The distribution of DETEs per family from RNA-seq analysis. (**c**) The number of upregulated elements per TE family in the prefrontal cortex of ASD individuals. The top TE family with the highest number of upregulated elements is shown on the left y-axis. The number of total elements in each TE family is shown on the right y-axis. Red bars represent Alu elements, blue bars represent LINE elements, and gray bars represent MIRs.

To characterize the TE expression profile in the prefrontal cortex of ASD individuals, we performed a transcriptomic study using postmortem brain tissues from ASD and unaffected individuals, as described in the Methods. We selected one (Liu’s study) from a total of four studies (Table S1) because it provides RNA-seq data on SRA deposits and has the largest sample size, including 21 males with ASD and 21 sex- and age-matched unaffected controls (female data were excluded in this study to remove sex bias). A total of 76,877 detectable TEs (excluding TEs with low expression) were used for differential expression analysis. This TE expression profiling revealed 864 differentially expressed TEs (DETEs) at the statistical threshold (adjusted *p* < 0.01, log_2_FC > 1) (Figure 2a, Table S2), including 218 downregulated and 646 upregulated DETEs. The distribution of upregulated DETEs per family showed that the majority of upregulated TEs belong to the Alu (48% or 308 elements) and LINE (24% or 154 elements) families (Figure 2b). Approximately 7% (or 47) and 21% (or 137) of the DETEs are members of the mammalian interspersed repeat (MIR) and other TE families, respectively. Moreover, the downregulated DETEs were distributed across other TE families, including LINE-1 (33%), MIRs (27%), and others (33%).

TEs are abundant in the human genome; thus, to ensure that the large number of DETEs of each family was not caused by this, we compared the number of DETEs of each family with the total number of elements in the family (Figure 2c). Interestingly, when compared with other TEs (LINE and MIR families), young families (AluY and AluS) exhibited more DE-Alu elements than the total number elements in the family. For the LINE family, most DE-LINEs belonged to the inactive family (L2 and L3), with a larger number of total elements than DE-Alu elements. These DETEs indicate epigenetic alteration at TE loci in the prefrontal cortex of ASD individuals. Moreover, only a small number of DE-LINE-1 (L1) elements, which is an active LINE family, were differentially expressed. However, the most active L1 subfamily (L1H) was not significantly expressed in the prefrontal cortex of ASD individuals in our analysis. In this study, we focused on the Alu family, which is a major DETE according to the number of DE-Alu elements and the total number of elements in the family.

### 2.2. Correlations between Alu Expression and Differentially Expressed Genes in the Prefrontal Cortex of ASD Individuals in Cis- and Trans-Regulatory Manners

**Figure 3:**
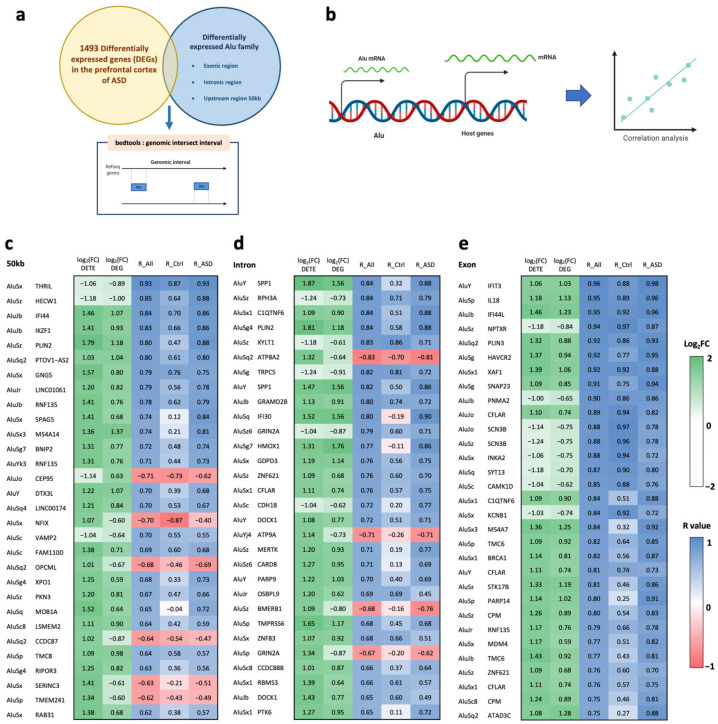
Correlation between differentially expressed Alu family members and their host DEGs in the prefrontal cortex of ASD individuals: (**a**) The intersection of genomic intervals between DE Alu and DEGs to identify host DEGs of individual DE-Alu elements classified into 50 kb upstream regions, intronic regions, and exonic regions created with BioRender.com (accessed on 4 January 2023). (**b**) Illustration of dysregulation of the Alu family affecting host/nearby gene expression created with BioRender.com (accessed on 4 January 2023). Heatmap (log_2_FC and R-value) of the top 30 DEGs most significantly correlated with the Alu family, as classified into 50 kb upstream (**c**), intronic (**d**), and exonic (**e**) regions.

Because TEs can provide enhancer elements, act as alternative promoters, and epigenetically regulate nearby and distant target genes, we performed correlation analysis between the expression of DETEs and differentially expressed genes (DEGs). We hypothesized that a dysregulation of TEs may reflect a change in an epigenetic mark that induces the transcriptome profile in the brains of ASD individuals. In this study, we performed Pearson’s correlation analysis for cis- and trans-regulation. We also reanalyzed the same RNA-seq data for differential gene expression analysis. To determine whether DETEs regulate the expression level of genes located near DETEs, we overlapped the genomic locations of DETEs and RefSeq genes to identify targeted genes using bedtools prior to correlation analysis (Figure 3a,b). First, DETE loci were classified based on genomic features, including regions upstream of the TSS (50 kb, 10 kb, and 1 kb), intronic regions, and exonic regions. We found 18,045 DETE positions located within or near 1587 locations corresponding to 1493 DEGs in the prefrontal cortex of ASD individuals compared to unaffected controls (Table S3). Most DETEs are located in the 50 kb upstream region (8199 loci) and intronic region (7232 loci) of 507 and 744 DEGs, respectively (Table 1). Correlation results showed that 534 DETE loci correlated significantly with DEGs in the prefrontal cortex of ASD individuals (Benjamini–Hochberg (BH)-adjusted *p* < 0.05). As expected, most of the correlating DETEs are located in the 50 kb and intronic regions of the DEGs. When the correlation was performed separately for ASD and unaffected individuals, we observed a different number of correlating DEGs. In ASD, a high number of DEGs correlated with DETEs, especially in the Alu family. When focusing on Alu elements, we found a greater difference in the number of significantly correlating DEGs between ASD and unaffected controls for 659 loci corresponding to 456 DEGs (Table 1, Table S4). In ASD, 161 Alu elements correlated with DEGs; in unaffected individuals, 86 Alu elements correlated with DEGs. A correlation matrix was generated that showed the difference in the top 30 significant loci between ASD and unaffected individuals (Figure 3c–e). These findings indicate that DEGs in the prefrontal cortex of ASD individuals may be associated with dysregulation of the Alu family.

For trans-regulation analysis, we performed correlation analysis between all probabilities between 659 DE-Alu and 1493 DEGs. A ~500 k correlation probability between DE-Alu was tested, and most of the DEGs showed a significant correlation with these elements (Table 2) based on Pearson’s correlation analysis with multiple hypothesis correction (FDR = 0.05). We found 40,599 significant probabilities that Alu influences 1465 DEGs in the prefrontal cortex of ASD individuals and 7141 probabilities for 1239 DEGs in unaffected individuals. These findings suggested that Alu elements contribute to the trans-regulation of several genes in the genome. This was expected, because TEs contribute to normal neurodevelopment and it is possible that these elements control other genes in the genome in addition to the DEGs.

To fully comprehend how TEs contribute to ASD, it may not be adequate to focus only on the number of DEGs involved in trans-regulation; the abundance of TE components in ASD and unaffected individuals is also interesting. Alu elements were found in higher abundance in ASD individuals than in unaffected controls, even though the results showed a similar number of DEGs in trans-regulation (316 and 196 elements, respectively). We thus overlapped significant probability loci between ASD and unaffected individuals (Figure S1), with 191 Alu elements found in both ASD and unaffected individuals (only 5 Alu elements from among the 196 found were exclusively in unaffected controls). These elements might be normal Alu elements that contribute to normal processes in the genome. Additionally, we discovered 125 Alu elements to be substantially associated in individuals with ASD but not in unaffected controls. These findings suggest that trans-regulatory elements from Alu are increased in the prefrontal cortex of ASD, leading to the dysregulation of the related genes.

### 2.3. The Alu-Mediated Gene Regulatory Network in the Prefrontal Cortex of ASD Individuals Is Associated with ASD and the Cell Death and Survival of Neural Cells

**Figure 4:**
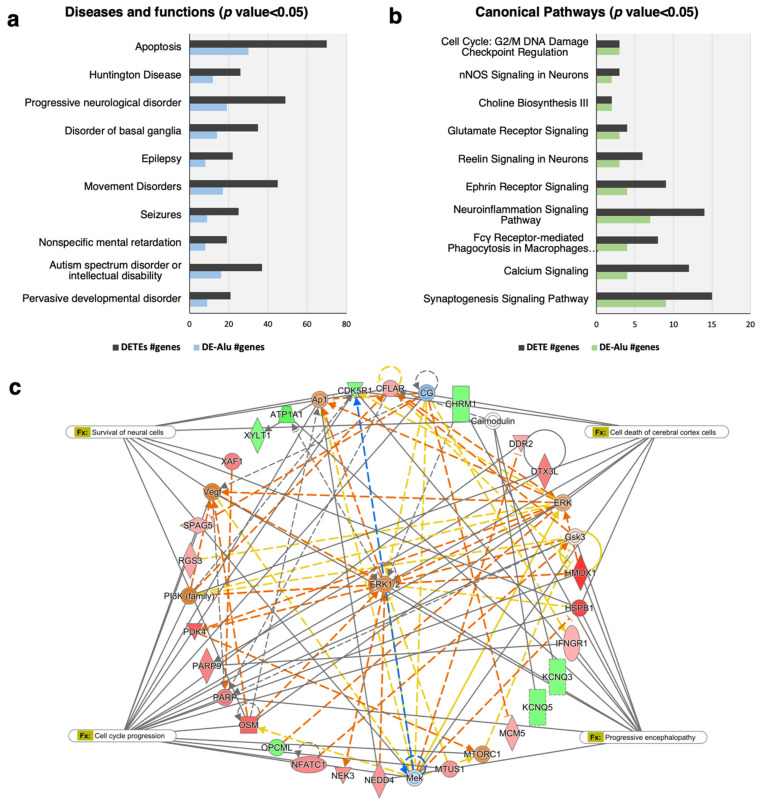
Functions and pathways of DEGs significantly correlating with DE-Alu elements in the prefrontal cortex of ASD individuals predicted by IPA software: (**a**) Diseases and functions associated with DEGs. (**b**) Canonical pathways associated with DEGs. (**c**) The gene regulatory network associated with DEGs was generated using IPA software. Red indicates upregulated genes and green indicates downregulated genes.

To determine whether DEGs in the prefrontal cortex of ASD individuals are associated with ASD and neuropathology, we obtained a list of significantly correlating DEGs for biological function and pathway analyses using IPA software (161 Alu-correlating loci corresponding to 133 DEGs in Table 1). The results showed that Alu-correlating DEGs were enriched for diseases and functions associated with ASD (16 genes, *p* = 1.19 × 10^−3^) and comorbid disorders, including pervasive developmental disorder, mental retardation, movement disorders, and epilepsy (Figure 4a, Table S5). We also found that Alu-correlated DEGs are related to apoptosis (30 genes, *p* = 5.75 × 10^−4^), and the gene regulatory network predicted by IPA also highlighted the survival of neural cells and the cell death of cerebral cortex cells (Figure 4c). Furthermore, IPA results showed the association of Alu-correlated DEGs with canonical pathways such as the synaptogenesis signaling pathway, neuroinflammation signaling pathway, cell cycle, and glutamate receptor signaling (*p* < 0.05) (Figure 4b, Table S6). These findings indicate that in the prefrontal cortex of ASD individuals, a dysregulation of Alu retrotransposons affects the expression of host/nearby protein-coding genes associated with the neuropathology implicated in ASD.

In addition to the predicted biological functions and pathways, we intersected the list of significantly correlating DEGs with autism candidate genes from the SFARI database using hypergeometric distribution analysis (Figure S2a). A Venn diagram revealed that 35 genes from among 309 DEGs correlated with DETEs were significantly enriched among autism candidate genes (*p* = 3.67 × 10^−6^); the list of overlapping genes associated with Alu elements is shown in Figure S2b. This finding suggests that DE-Alu influences autism gene expression in the prefrontal cortex, which may lead to risk of ASD.

### 2.4. Consensus Sequence and Transcription Factor Binding Sites at DE-Alu Elements

**Figure 5:**
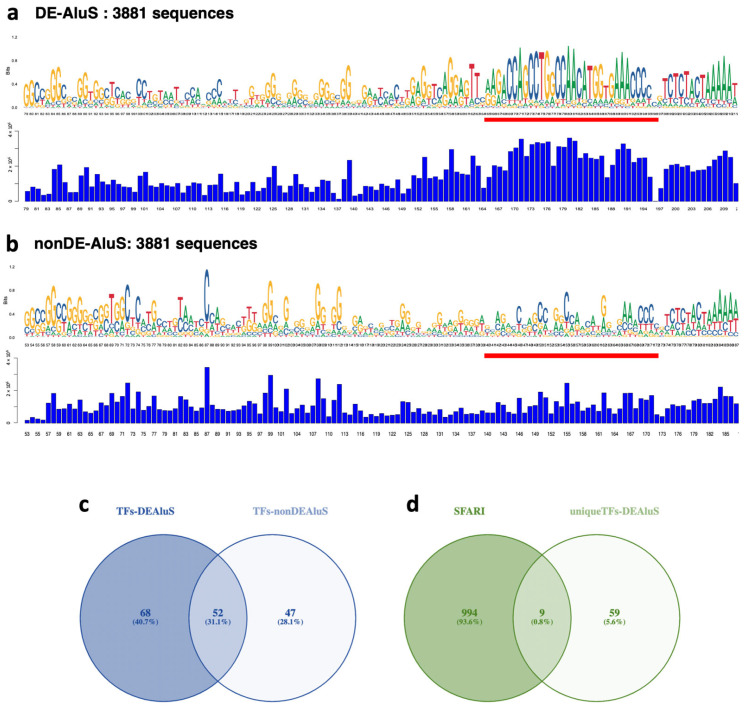
Consensus sequences and conservation scores of DE-AluS and non-DE-AluS. This figure shows the consensus sequence of the AluS family at the left arm of elements (containing the promoter region) of DE-AluS (**a**) and non-DE-AluS (**b**). Red lines highlight a conserved region for DE-Alu compared with non-DE-Alu. (**c**) A Venn diagram between predicted transcription factors binding to DE-AluS and non-DE-Alu. (**d**) A Venn diagram between unique transcription factors binding to DE-AluS and SFARI genes.

Although Alu elements are abundant in the genome, only a small portion of Alu elements are active and escape from restriction by an epigenetic mechanism. To determine the characteristics of the DE-Alu sequence, we conducted multiple sequence alignment analysis and identified the consensus sequence from each DE-Alu compared with nondifferentially expressed elements (non-DE) by random selection. To draw the closest comparison between DE-Alu and non-DE-Alu, we randomly chose the non-DE-Alu sequence equally to the DE-Alu sequence (3881). This type of selection with similar numbers (~5000) has been performed in a previous study [28]. We performed an analysis of the AluS and AluY families, which are the major DETEs in the RNA-seq results. The analysis focused on the left arm monomer of Alu elements, which is the conserved region and contains promoters. A comparison between the consensus sequence and the conservation score indicated by the sequence logo and bar chart, respectively, showed a highly conserved region in the DE-AluS family compared with non-DE-AluS. The red lines in Figure 5a,b indicate higher conservation. AluY showed a slightly conserved region between DE-AluY and non-DE-AluY (Figure S3). This difference may be the reason for the abnormal expression of DETEs, as controlled by upstream regulators that ultimately affect downstream genes.

We next predicted transcription factor binding sites at DE-AluS and non-DE-AluS consensus sequences using the JASPAR CORE database. We selected only the conserved region for scanning using 951 matrix profiles corresponding to 681 TFs (filtered by *Homo sapiens*) and found 191 matrix profiles (120 TFs) with high relative scores (relative score thresholds = 0.8) in the DE-AluS and 243 matrix profiles (99 TFs) in non-DE-AluS. When overlapping TFs between DE-AluS and non-DE-AluS, we detected 68 unique TFs binding to the DE-AluS consensus sequence (Table 3, Figure 5c). We further identified unique TFs associated with ASD by overlapping with ASD candidate genes from the SFARI database (Figure 5d). In total, 12 TFBSs within the DE-AluS consensus sequence corresponding to nine ASD-associated TFs (Table S7), including NR2F1, VDR, NR4A2, NR1D1, RORA, SATB1, ARNT2, NR4A2, AR, TCF7L2, and NR4A2, were found. These findings provide a better understanding of the upstream regulators of DE-Alu elements associated with ASD.

### 2.5. DNA Methylation of Alu Is Altered in the Prefrontal Cortex of ASD Individuals

We conducted a pilot study using human postmortem brain tissues of ASD and unaffected controls that were obtained from the Harvard Brain Tissue Resource Center, consisting of 13 samples of prefrontal cortex tissues from ASD individuals (*n =* 7) and unaffected controls (*n =* 6). There were no significant differences in age, sex, or postmortem interval (PMI) between the ASD group and the unaffected control group. Our postmortem ASD brain tissue was matched with the ASD sample used for RNA-seq reanalysis using brain IDs, and we applied the COBRA method to determine the global DNA methylation of the AluSx subfamily, which is the most abundant Alu element in the genome. The COBRA method was designed to measure DNA methylation levels (^m^Cs) and patterns (^m^C^m^C, ^m^C^u^C, ^u^C^m^C, ^u^C^u^C) at AluSx subfamily promoters according to our previous study [20]. The percentage of AluSx methylation levels and patterns from the COBRA analysis are shown in Table 4. The percentages of partially methylated patterns (%^u^C^m^C) were significantly increased in ASD compared with sex- and age-matched controls (∆%^u^C^m^C = 0.285, *p* = 0.037) (Table 4). Due to the heterogeneity of ASD, we classified ASD individuals based on the ADI-R score for verbal-ASD (V-ASD) and nonverbal ASD (NV-ASD), which were obtained from multiple ASD studies (Table S1). Interestingly, we found differences in AluSx methylation patterns in the V-ASD individuals compared with the sex- and age-matched controls, including total methylation (∆%^m^C = −1.159, *p* = 0.019), hypermethylated patterns (∆%^m^C^m^C = −2.650, *p* = 0.004), and partially methylated patterns (∆%^u^C^m^C = 0.462, *p* = 0.023; ∆%^m^C^u^C = 1.225, *p* = 0.031); however, we did not observe AluSx methylation changes in the NV-ASD individuals compared with the sex- and age-matched controls. Because changes in the DNA methylation level of Alu elements were observed, we also determined the expression levels and relative copy numbers of AluSx elements in postmortem brain tissues.

**Figure 6:**
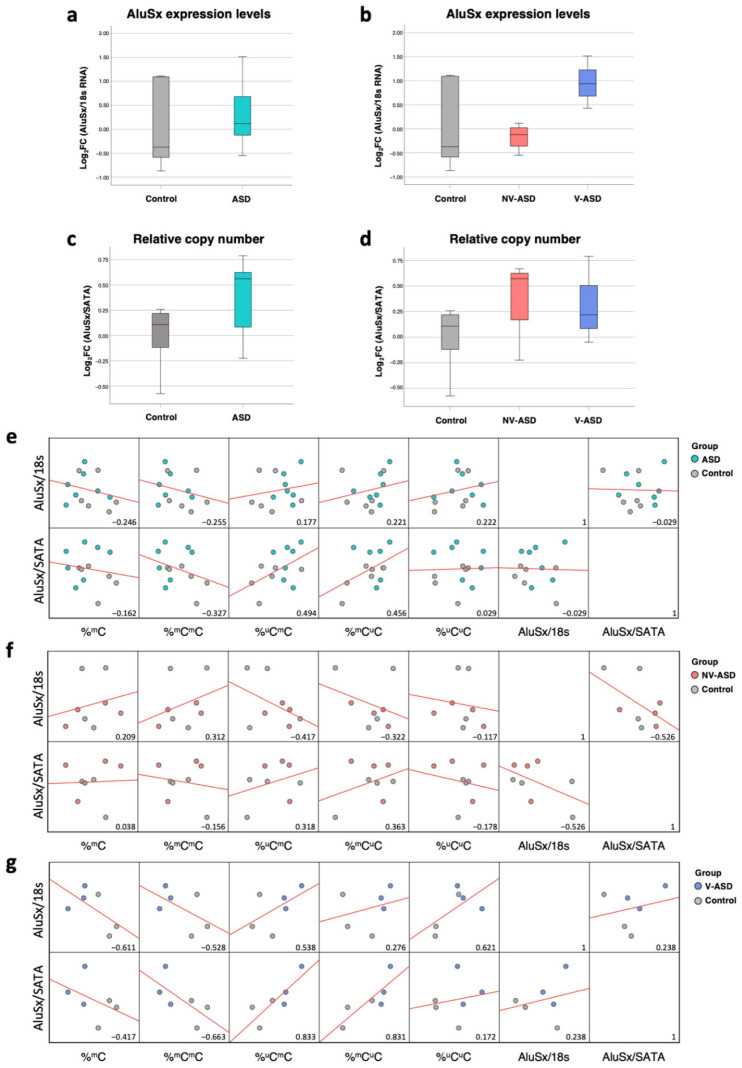
AluSx expression level and relative copy number in the prefrontal cortex of ASD individuals: (**a**) Box plot of AluSx expression levels in ASD and matched controls. (**b**) Box plot of AluSx expression levels in ASD subtypes (NV-ASD and V-ASD) and matched controls. (**c**) Box plot of relative AluSx copy number in ASD and matched controls. (**d**) Box plot of relative AluSx copy number in ASD subtypes (NV-ASD and V-ASD) and matched controls. (**e**) Correlation matrix between DNA methylation, expression, and copy number of AluSx in ASD and matched controls. (**f**) Correlation between expression level and relative copy number and DNA methylation of AluSx in NV-ASD. (**g**) Correlation between expression level and relative copy number and DNA methylation of AluSx in V-ASD. Red lines in (**e**–**g**) represent trend lines (linear model).

There was no statistically significant difference between ASD individuals and matched controls with regard to AluSx expression level or relative copy number (Figure 6a,c) or among ASD subtypes based on ADI-R scores (Figure 6b,d); however, correlation analysis revealed an association between DNA methylation and AluSx activity in the prefrontal cortex of ASD individuals and unaffected controls (Figure 6e–g). The correlation between partially methylated patterns (%^u^C^m^C and %^m^C^u^C) that contained one unmethylated CpG showed a moderate positive correlation with AluSx copy number (R = 494 and R = 456, Figure 6e). The correlation between DNA methylation and expression of AluSx showed a weak correlation in the ASD and unaffected individuals (R = 0.177). Additionally, correlation analysis involving the ASD subtype revealed that significant AluSx methylation levels and patterns showed moderate to high correlation levels with AluSx expression and relative copy number in the V-ASD subtype (Pearson’s correlation; |R| ranges = 0.417–0.833) (Figure 6f,g). These findings indicate that DNA methylation of AluSx, which is altered in the prefrontal cortex of ASD individuals, contributes to Alu expression and copy number in prefrontal cortex tissues.

### 2.6. Correlation Analysis between ADI-R Score and DNA Methylation and Expression of Alu Elements in ASD Brain Tissues

**Figure 7:**
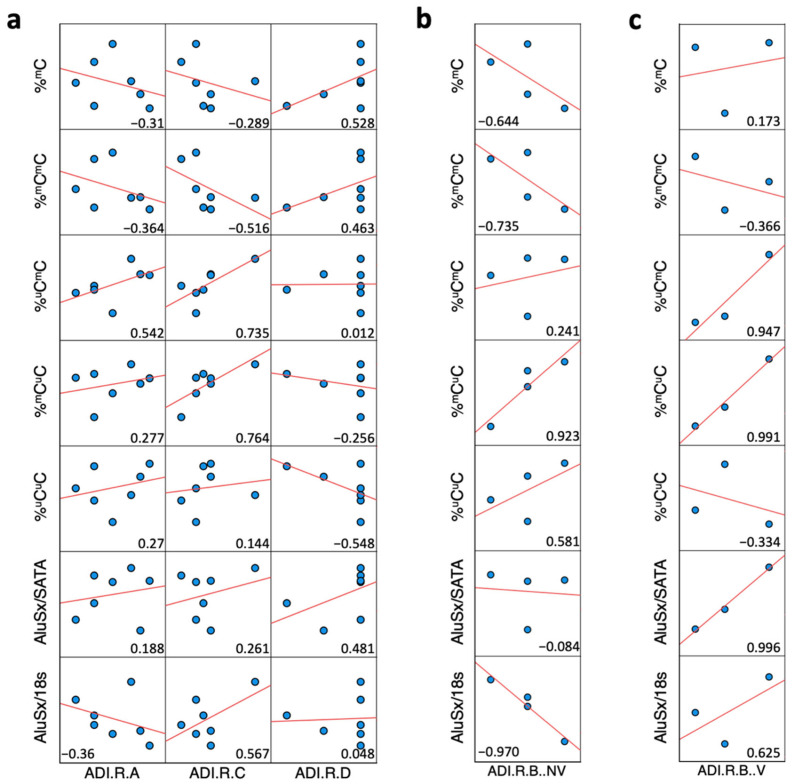
Correlation matrix between ADI-R score and DNA methylation, expression, and relative copy number of Alu elements in the prefrontal cortex: (**a**) Correlation between Alu elements and ADI-R A, social interaction; ADI-R C, restricted, repetitive, and stereotyped patterns of behavior; and ADI-R D, abnormality of development evident at or before 36 months. (**b**) Correlation between Alu elements and ADI-R B; nonverbal communication. (**c**) Correlation between Alu elements and ADI-R B; verbal communication. Red lines represent trend lines (linear model) and blue points represent ASD individuals.

To determine whether Alu elements are related to ASD symptoms, we correlated the DNA methylation, expression, and relative copy number of Alu elements with the ADI-R scores of ASD individuals. First, the correlation between ADI-R and Alu elements in the prefrontal cortex revealed the Alu methylation level and pattern (%^m^C and %^m^C^m^C) to be related to worse ADI-R scores, especially in the repetitive behavior domain (ADI-R C) and the communication domain for nonverbal individuals (ADI-R B NV). Partially methylated patterns (%^m^C^u^C and %^u^C^m^C) correlated positively with ADI-R (ADI-R C, ADI-R B NV, and ADI-R B V). Moreover, the expression and relative copy number of Alu elements correlated highly with the communication domain for both NV and V (ADI-R B NV and ADI-R B V) (Figure 7). These findings indicate that Alu elements may be related to ASD symptoms based on ADI-R scores and deserve further study using animal models and behavioral examinations.

### 2.7. Confirmation of Expression Levels of DEGs Associated with Alu Elements in Postmortem Brain Tissues

**Figure 8:**
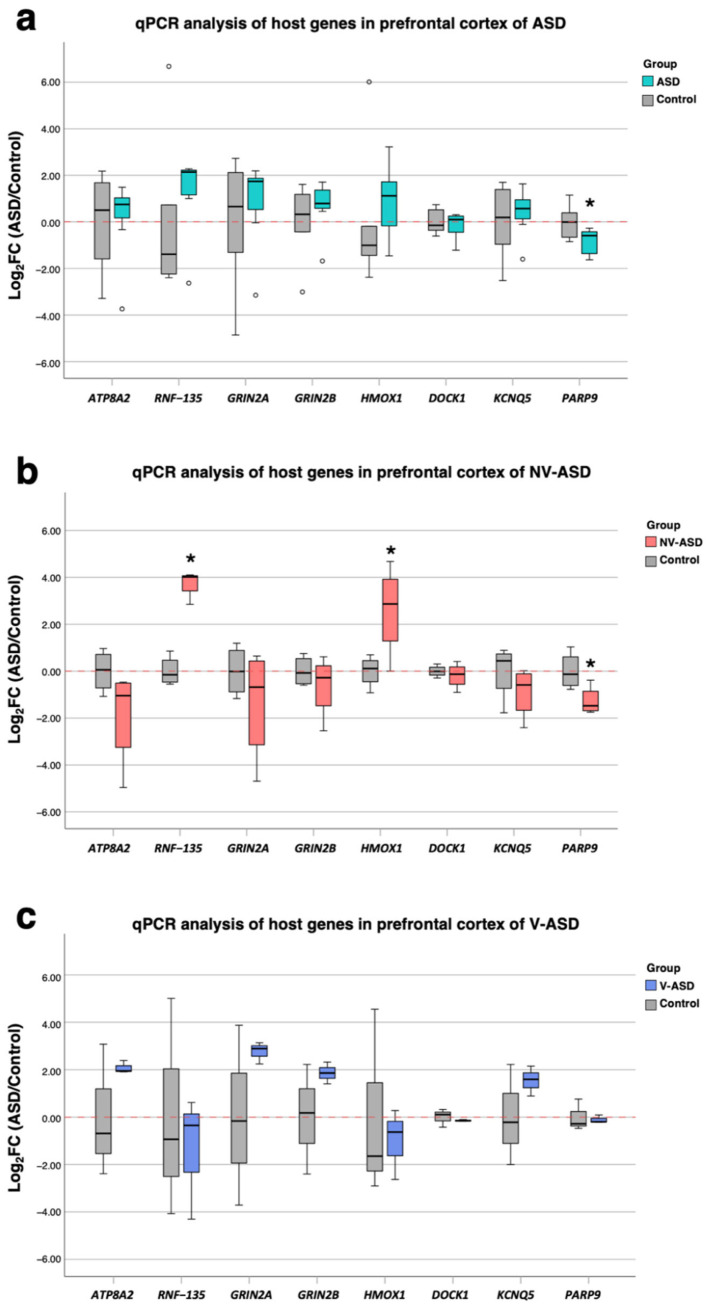
Quantitative RT–PCR analysis of selected DEGs significantly correlating with DE-Alu elements: (**a**) Box plot of the comparison between ASD (*n =* 7) versus unaffected (*n =* 6) individuals. (**b**,**c**) Box plot of the comparison between ASD subtype (NV-ASD and V-ASD) versus sex- and age-matched unaffected individuals. Dots represent outliers and * *p* < 0.05.

To confirm the expression of significantly correlated DEGs, we performed qPCR analyses using our postmortem brain tissues of ASD (*n =* 7) and unaffected (*n =* 6) individuals. We selected DEGs from the most significantly correlating DEGs or IPA results associated with the neuropathology of the cortex (i.e., *ATP8A*, *RNF135*, *GRIN2A*, *GRIN2B*, *HMOX1*, *DOCK1*, *KCNQ5*, and *PARP9*) (Table 5). The qPCR results showed significantly decreased *PARP9* gene expression in ASD compared with unaffected controls (Figure 8a) (log_2_FC = −0.87, *p* = 0.038). Moreover, we classified ASD individuals based on ADI-R B-for nonverbal (NV) and verbal (V) individuals as NV-ASD and V-ASD individuals. Expression of the *RNF135* and *HMOX1* genes was found to be significantly increased in NV-ASD individuals compared to sex- and age-matched controls (Figure 8b) (*RNF135*: log_2_FC = 3.75, *p* < 0.01, and *HMOX1*: log_2_FC = 2.60, *p* = 0.045). There were no significant genes observed in V-ASD compared with sex- and age-matched controls (Figure 8c). These results show the reproducibility of DEGs associated with Alu elements in both RNA-seq data and our postmortem brain tissues.

### 2.8. RNF135 and HMOX1 Genes Are Related to Neuron Number in the Prefrontal Cortex

**Figure 9:**
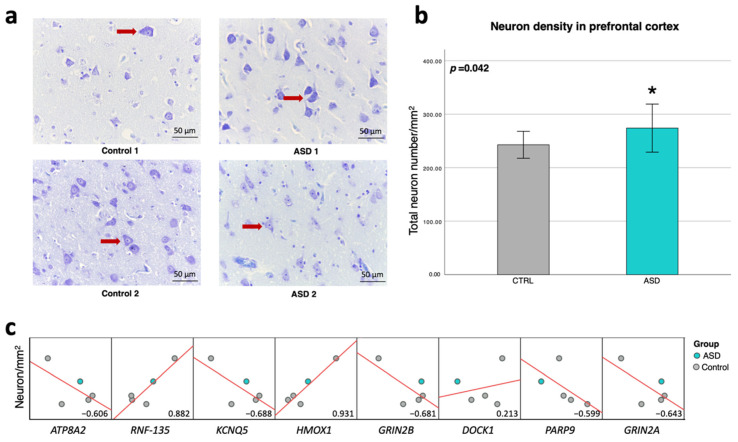
Neuronal cell density and correlation with DEGs in the prefrontal cortex: (**a**) Representative images of Nissl staining (red arrows represent neurons). (**b**) Total neuron number/mm^2^ in the prefrontal cortex of ASD (*n =* 3) and unaffected (*n =* 5) individuals. (**c**) Correlation analysis (R value) between the expression level of DEGs from qPCR analyses and neuron density (red lines represent trend lines; linear model). * *p* < 0.05.

According to biological function and gene regulatory network analyses, we found that DEGs correlating with Alu elements are associated with the survival of neural cells and the cell death of cerebral cortex cells. We hypothesized that Alu elements may be associated with genes involved in the neuropathology found in the postmortem brain tissues of ASD individuals. Total neuron density was quantified in prefrontal cortex tissues (ASD; *n =* 3, unaffected; *n =* 5) stained with the Nissl staining method (Figure 9a). According to this analysis, neuron density in the prefrontal cortex between ASD and unaffected individuals was significantly increased in the former (*p* = 0.042) (Figure 9b). We next performed correlation analysis between neuron density and the expression level of DEGs associated with Alu elements using Pearson’s correlation (Figure 9c), with a moderate to high correlation (|R| = 0.599–0.931) with neuron density in the prefrontal cortex, except for *DOCK1* (R = 0.213). In particular, the *RNF135* and *HMOX1* genes, which were significantly differentially expressed, as confirmed by qPCR, showed a higher correlation with neuron density than the other genes. It is important to note that there was a limitation regarding the number of paraffin-embedded tissues available, especially for matching with frozen tissues for qPCR analyses. Thus, we did not analyze ASD individuals by classifying them into NV-ASD and V-ASD groups. Further study using a larger sample size is needed.

### 2.9. Locus-Specific DNA Methylation of AluYk3 Located in the Upstream Region of the RNF-135 Gene

**Figure 10:**
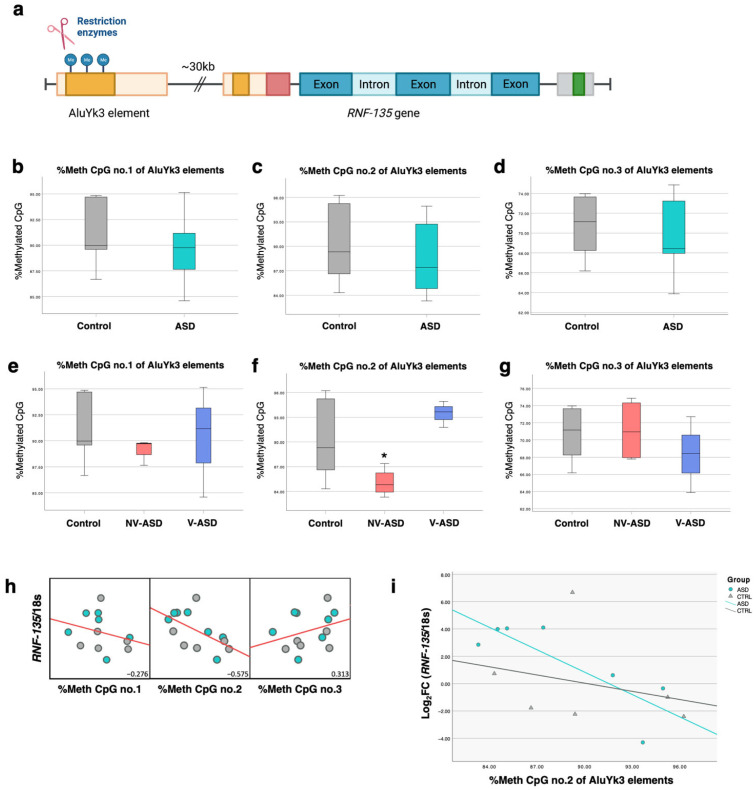
Locus-specific DNA methylation of AluYk3 located near the *RNF-135* gene: (**a**) Illustration of AluYk3 elements located upstream of the *RNF-135* gene. Three CpG sites in AluYk3 were determined using the COBRA method created with BioRender.com (accessed on 20 January 2023). (**b**–**d**) Box plots of AluYk3 methylation at three CpG sites in ASD and unaffected individuals. (**e**–**g**) Box plots of AluYk3 methylation at three CpG sites in ASD subtypes (NV-ASD and V-ASD) and unaffected individuals. (**h**) A correlation matrix between AluYk3 methylation and expression of *RNF-135* (red lines represent trend lines; linear model) (**i**) Scatter plots between AluYk3 methylation (CpG no. 2) and expression of *RNF-135* in ASD and unaffected individuals. * *p* < 0.05.

**Figure 11:**
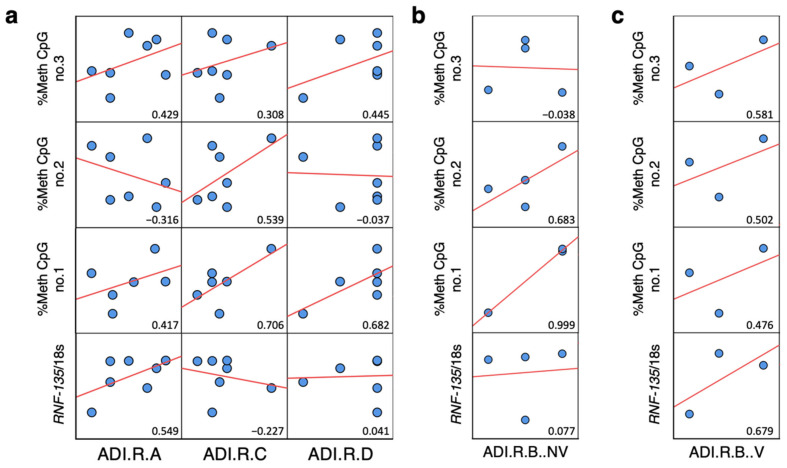
Correlation matrix between ADI-R scores, AluYk3 methylation, and *RNF-135* expression in the prefrontal cortex: (**a**) Correlation between AluYk3 elements, the *RNF-135* gene, and ADI-R A; social interaction, ADI-R C; restricted, repetitive, and stereotyped patterns of behavior, and ADI-R D; abnormality of development evident at or before 36 months. (**b**) Correlation between AluYk3 elements, the *RNF-135* gene, and ADI-R B; nonverbal communication. (**c**) Correlation between AluYk3 elements, the *RNF-135* gene, and ADI-R B; verbal communication. Red lines represent trend lines (linear model) and blue points represent ASD individuals.

To investigate the susceptibility loci of Alu elements in DEGs in brain tissues, we applied the COBRA method to assess locus specificity at three CpG sites of AluYk3 elements located upstream of the *RNF-135* gene (Figure 10a). As in the previous analyses, we compared methylation levels and patterns between all ASD, NV-ASD, and V-ASD individuals versus sex- and age-matched controls. The results showed no significant differences in AluYk3 methylation between all ASD individuals versus unaffected individuals (Figure 10b–d, Table S8). Interestingly, we found one differentially methylated CpG position at AluYk3 elements, whereby the CpG second position was hypomethylated in the NV-ASD group (∆%methylated CpG no.2 = −6.79, *p* = 0.033) but not in all ASD or V-ASD groups compared with sex- and age-matched controls (Figure 10e–g). We performed correlation analysis between AluYk3 methylation and *RNF135* expression levels and found that the %methylated CpG second position at AluYk3 showed a negative correlation with *RNF135* (R-value = −0.575) (Figure 10h,i). These findings suggest that AluYk3 located ~30 kb upstream of the *RNF135* gene might contribute to the regulation of *RNF135* gene expression levels in the prefrontal cortex of ASD individuals via DNA methylation. Nevertheless, we did not detect any significant difference in other selected Alu elements located in other DEGs, such as the *HMOX1* gene (Table S8). Notably, it is possible that other CpGs or other epigenetic mechanisms of these Alu elements may have contributed to host DEG expression. Due to the limitation of the COBRA method, only 1-3 CpG sites at the promoter region of Alu elements were detected for these analyses. We also performed correlation analysis between AluYk3 methylation and the ADI-R scores of ASD individuals. Differentially methylated CpG second positions showed moderate correlations with ADI-R scores, including ADI-R C (restricted, repetitive, and stereotyped patterns of behavior domain, R = 539) and ADI-R B (nonverbal and verbal communication, R = 0.683 and 0.502) (Figure 11a–c).

## 3. Discussion

At present, it is not known exactly what causes abnormal gene expression levels in postmortem brain tissues of ASD individuals. Most analyses of DNA methylation and transcriptome studies in postmortem brain tissues of ASD individuals have focused on protein-coding regions rather than noncoding regions, with the latter contributing most of the CpG sites in the human genome, including TEs. TEs can be classified into two types based on their structure and the requirement of reverse transcription for transposition: DNA transposons and retro (RNA) transposons [17]. There are two main types of retrotransposons: long terminal repeats (LTRs: ERV) and non-long terminal repeats (non-LTRs), and most retrotransposons in the human genome are the latter [17,29]. The most common non-LTR retrotransposons are long interspersed nuclear element 1 (LINE-1 or L1) and short interspersed nuclear element (SINE: Alu and MIR) transposons, which remain active and comprise approximately 30% of the human genome at a copy number of over 1 million elements [17,29]. Dysregulation of TEs can affect developmental processes via their gene product through new insertions that cause genetic changes. Moreover, retrotransposons introduce a promoter or premature terminator (poly-A site) into host genes, causing transcriptional disruption. Retrotransposons can also act as enhancers of host/nearby genes because they contain several transcription factor binding sites [17].

Several pieces of evidence suggest that Alu elements and other TEs influence mammalian genome evolution by introducing novel gene formation, transcriptome diversity, and transcriptional regulatory elements [18,30]. Alu elements are beneficial for the development of the central nervous system, including brain formation and function [30,31]. Additionally, previous studies found that Alu controls several genes that are important for neuronal function, such as the serotonin transporter gene [32,33,34]. However, a loss of control of Alu element activity by epigenetics, due to environmental and other nongenetic factors, contributes to many human diseases, including neurological diseases [30]. This evidence supports the idea that epigenetic dysregulation in ASD brain tissues may modulate the activities of Alu elements and alter the transcriptome.

In this study, we characterized the TE expression profile of the prefrontal cortex of ASD individuals. We found differentially expressed TEs from the retrotransposon family, including the Alu, LINE, and MIR families (Figure 2b). Due to the high abundance of TEs in the human genome, we also considered the total number of elements in each family together with the number of differentially expressed elements (Figure 2c). Although we observed many dysregulated elements belonging to the LINE-2 (L2), LINE-3 (L3), and MIR families, it appears that the large number may be due to the total number of elements. Another interesting TE family is the dysregulated LINE-1 family (L1), which is an active family of LINEs. When we considered subfamilies of L1, dysregulated L1 was classified as belonging to the L1M subfamily, which is the oldest and least active family of L1 subfamilies [35]. The most active and youngest subfamily of L1 (L1H) was not detected in our analysis. This is consistent with a study by Shpyleva, who performed qPCR analysis of the L1H subfamily in ASD using the cerebellum and prefrontal cortex, with L1H being significantly overexpressed only in the former [25]. Indeed, we focused on the dysregulated elements of the Alu family because this TE family has a large number of dysregulated elements compared with the total number of Alu and other TE families. Moreover, the highest number of dysregulated Alu belonging to the AluSx and AluY subfamilies constitutes a middle and young subfamily of Alu elements [35].

We further investigated how this Alu dysregulation affects ASD neuropathology. At present, our understanding of the precise role of Alu elements in ASD development remains limited. Their possible function may be as a result of LINE-1 and Alu elements acting as enhancers or promoters for host genes [17]. Alu retrotransposons are mostly distributed in gene-rich regions close to the TSS of genes [36,37] and contain many transcription factor (TF) binding sites [38]. According to the study of Su and colleagues in 2014, Alu retrotransposons are significantly conserved at the upstream region of genes and enriched for enhancer-like epigenetic regulation [26]. Furthermore, the epigenetic state of Alu retrotransposons located upstream of genes might regulate the expression and chromatin structure of genes associated with the cell cycle [39]. In our study, we expected that dysregulation of the Alu family would reflect epigenetic alterations at Alu elements, which may impact host/nearby gene expression. We thus performed correlation analysis between the expression of Alu and their host genes. From 1493 DEGs in the prefrontal cortex of ASD individuals, as many as 161 loci corresponding to 133 DEGs correlated significantly with Alu elements located within/nearby host DEGs. The biological function of these genes regulated by Alu is associated with ASD and comorbid disorders. In addition to canonical pathway analyses, these correlating DEGs were significantly enriched in the synaptogenesis signaling pathway, neuroinflammation signaling pathway, cell cycle, and glutamate receptor signaling. Accumulating evidence suggests that a deficiency of genes in pathways involving synaptic protein synthesis and degradation, postsynaptic scaffold architecture, and neurotransmitter receptors contribute to synapse deficits in ASD individuals [40]. Moreover, several studies have demonstrated that children with ASD experience immune system changes at both systemic and cellular levels. In fact, individuals with ASD exhibit signs of active inflammation and altered gene expression in immune signaling and functional pathways [41]. Accordingly, we investigated how these Alu elements are dysregulated and affect gene activity in the brain tissues of ASD individuals.

In this study, we found that DE-AluS elements have a conserved region within the left arm monomer (promoter) when compared with non-DE-AluS. This suggests that the conserved region may contribute to a dysregulation of DE-AluS in ASD. We also evaluated transcription factor binding sites at the conserved region of DE-AluS in the prefrontal cortex of ASD individuals. Interestingly, we found that nine TFs binding to this region comprise autism candidate genes in the SFARI database. An interesting TF is retinoic acid-related orphan receptor alpha (RORA), a nuclear hormone receptor which is downregulated in both brain tissues and lymphoblastoid cell lines from individuals with ASD versus controls [42]. Interestingly, RORA not only transcriptionally regulates the aromatase enzyme, which converts testosterone to estrogen, but is itself under both negative and positive feedback regulation by male and female sex hormones, respectively [42]. These observations led Sarachana et al., (2011) to suggest that a decrease in RORA and its transcriptional target aromatase may be related to the reported elevated testosterone in amniotic fluid associated with ASD traits, which has been proposed as a factor influencing the male sex bias in ASD [43]. Sex differences in TEs in the human genome may be related to the biology of sex differences, such as higher TE density on the Y chromosome, differences in DNA methylation regulators (DNA methyltransferase enzymes and methylcytosine dioxygenases; TET), and histone modification marks [44]. Another interesting TF is vitamin D receptor (VDR), a nuclear receptor/steroid hormone receptor superfamily. VDR regulates several targeted genes involved in cellular proliferation and differentiation [45]. Genetic polymorphisms in *VDR* are associated with risk of ASD [46]. There is evidence that approximately 50% of children with ASD have vitamin D deficiency and that 30% have insufficient vitamin D, with serum vitamin D correlating negatively with ASD severity [47]. In the brain, vitamin D has several benefits, including calcium signaling and the synthesis of neurotrophic factors and neurotransmitters [48]. Our findings indicate that upstream regulators of TEs may be key factors for transcriptome diversity in ASD brain tissues, and further study is warranted.

Lowe et al., (2007) reported that retrotransposons are most often located in genes involved in development and transcriptional regulation, which may reflect that retrotransposons are involved in a common mechanism for development [49]. In addition to epigenetic alterations of retrotransposons at the integration site, including DNA methylation and chromatin remodeling, retrotransposon expression can be restricted and lead to the expression of host/neighboring genes [50]. Retrotransposon activity is suppressed through epigenetic mechanisms that silence their expression, and Alu hypomethylation has been reported in response to environmental exposures [51,52,53]. Current evidence links aberrant LINE-1 and Alu DNA methylation to human brain disorders, especially in patients with schizophrenia and ASD [54]. In the ASD brain, whole-genome sequencing analysis has revealed a higher number of novel insertions of LINE-1 and Alu elements than in normal brain tissues [24].

We have previously reported alterations in the global DNA methylation of LINE-1 and Alu retrotransposons in lymphoblastoid cell lines (LCLs) of ASD subtypes, and such alterations were specific to the ASD case subgroup based on clinical phenotype [20,21]. An alteration in LINE-1 methylation in LCLs was specific for the ASD subtype with severe language impairment, whereas an alteration in Alu methylation was specific for ASD with mild symptoms. Moreover, several genes with LINE-1 and Alu insertions were differentially expressed in the peripheral blood and LCLs of ASD. These genes are known to be associated with neurodevelopmental disorders. In the present study, we used COBRA to determine global AluSx methylation levels and patterns in the prefrontal cortex of ASD individuals. Although there was no significant difference in the overall methylation level (%^m^C) of the Alu promoter, we found that the percentage of partially methylated patterns containing one unmethylated CpG in the promoter was significantly increased (%^u^C^m^C) in all ASD individuals versus unaffected individuals. Interestingly, these methylation patterns exhibited moderate correlations with AluSx expression and copy number in the prefrontal cortex of ASD individuals.

When we classified ASD individuals based on the ADI-R score for nonverbal and verbal ASD, we found differences in the Alu methylation in the brain of individuals with each ASD subphenotype. For global Alu methylation, Alu methylation was changed specifically in the prefrontal cortex of V-ASD individuals. Correlation analysis between Alu methylation, expression, and relative copy number showed a high correlation in V-ASD; however, the Alu expression level and relative copy number did not differ between ASD individuals and unaffected controls, even when ASD individuals were subtyped. This is unnecessary for disrupting host gene expression because Alu elements or other TEs can provide regulatory elements for cis- and trans-regulation to interfere with Pol-II transcription, whereas Alu elements are transcribed by Pol-III [55]. Moreover, correlation analysis with the ADI-R scores of ASD individuals showed Alu elements to be associated with ASD symptoms. Based on this finding, the role of Alu elements in autism-related behaviors deserves further study.

In this research, we sought to identify autism-susceptibility loci of TEs in the brains of ASD individuals. We selected host DEGs and investigated the methylation levels of Alu elements located within or near host DEGs. First, we confirmed the expression level of host DEGs using qPCR and found that *HMOX1*, *PARP9*, and *RNF135* were differentially expressed in the prefrontal cortex of ASD individuals. Unfortunately, when we implemented the COBRA method to assess the locus-specific DNA methylation of Alu elements, we found only one differentially hypomethylated locus at AluYk3 elements. AluYk3 elements were found to be located ~30 kb upstream of the *RNF135* gene. *RNF135* was overexpressed in both RNA-seq data and qPCR analysis; interestingly, AluYk3 methylation correlated significantly with *RNF135* expression. This result suggests that AluYk3 may provide cis-regulatory elements for the *RNF135* gene located nearby. *RNF135*, or Ring Finger Protein 135 or RING-Type E3 Ubiquitin Transferase *RNF135* gene, is an autism candidate gene in the SFARI database involved in protein–protein and protein–DNA interactions. *RNF135* has been associated with ASD [56,57] and neurofibromatosis [58] and reported to be involved in intronic Alu-mediated recombination in NF1 patients [58]. Regardless, the role of *RNF135* in neurodevelopment remains unclear, though there is evidence showing that *RNF135* can promote the proliferation of human glioblastoma cells in vivo and in vitro [59]. We also observed that this region of pathogenic copy number variation contains several genes (*NF1*, *RNF135*, *ADAP2*, and *SUZ12*) and shows many Alu-mediated recombination events [58,60].

Finally, we sought to determine whether Alu elements are associated with neuropathology in the ASD brain. In our analysis, neuron density in the prefrontal cortex of ASD individuals was increased compared with unaffected individuals. Our correlation analysis with qPCR results showed that neuron number in the prefrontal cortex correlated highly with Alu-correlating genes. These findings suggest that Alu elements may contribute to neuropathology in the prefrontal cortex of ASD individuals by disrupting the gene expression involved in ASD and neuropathology, such as cell death and survival. The prefrontal cortex area is responsible for memory, verbal attention, and language processing, which are strongly linked to ASD [61,62,63]. Several studies have shown changes in cytoarchitecture in the brains of ASD individuals, such as neuron and glial cell number or density. Studies by Falcone et al. and Courchesne et al. also demonstrated increased neuron density in the prefrontal cortex of ASD individuals compared with unaffected controls [64,65]; however, there is inconsistency among studies with respect to the number of neurons in the cerebral cortex of ASD individuals [66,67]. It is very important to note that our finding is from a preliminary study highlighting the impact of TEs, especially Alu elements, on the neuropathology in the prefrontal cortex of ASD individuals.

Because many comparable sequences have low mappability, TE profiling is exceedingly difficult. The techniques used to prepare RNA libraries, requirements for certain parameters to address their mappability, tools used to detect their transcripts, and genomic locations can all have an impact on these limitations. Thus, some active young TEs may not have been detected in our analysis due to low mappability and low abundance. Moreover, our analysis did not detect the potential expression of TEs not in the hg38 reference genome. Other limitations include use of postmortem brain tissues, the small sample size because of the limited availability of brain tissues, no pilot study for calculating a suitable sample size, the characterization of methylated DNA-binding proteins, and functional consequences. There was also a limitation regarding the COBRA method used for DNA methylation analysis due to restriction sites of the enzyme, and the CpG sites determined in our study may not reflect the entire methylation status of the promoter of Alu elements.

**Figure 12:**
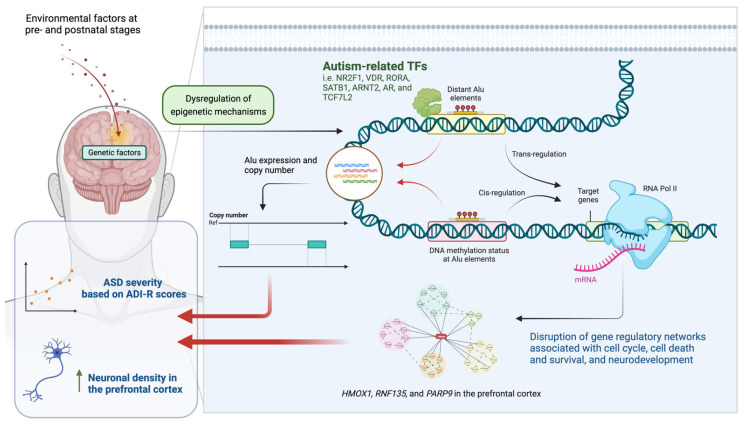
A schematic diagram illustrating a proposed mechanism by which Alu elements contribute to neuropathology in ASD brain tissues, as created with BioRender.com (accessed on 4 January 2023).

Based on our findings, we propose a molecular mechanism related to neuropathology in ASD brain tissues (Figure 12). Environmental risk factors for ASD induce a dysregulation of epigenetic mechanisms in the brain, leading to changes in the activities of Alu elements, as well as other TEs. The dysregulation of TEs contributes to the transcriptome through two possible mechanisms: (i) altering the transcriptome profile of the brain via cis- and trans-regulation of targeted genes and (ii) directly changing the transcriptome profile via functional products, including transcripts (that directly interfere with RNA) and DNA (which alters DNA sequence). Both mechanisms may be related to autism-associated TFs that lead to a disruption of the interactome, which is related to the neuropathology of ASD brain tissues.

## 4. Materials and Methods

**Figure 13:**
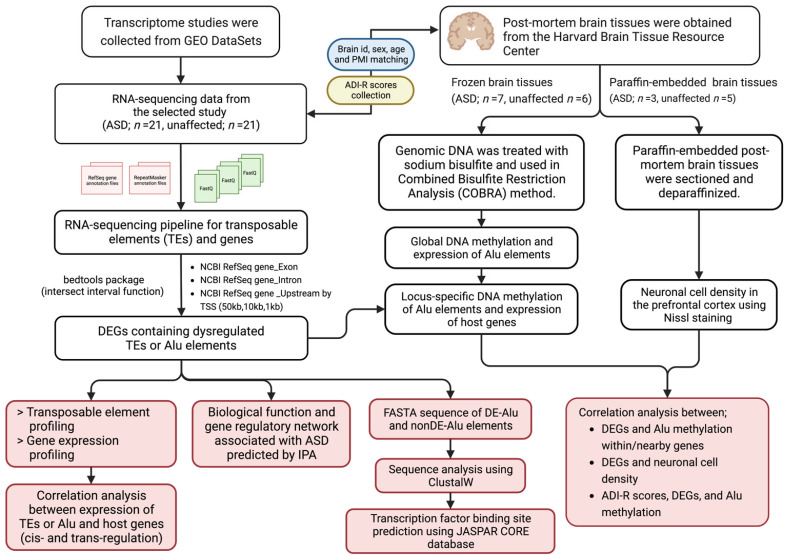
Experimental workflow of this study created with BioRender.com (accessed on 19 March 2023).

Figure 13 describes the overall strategy and experimental workflow of this study. To investigate relationships between TEs, the transcriptome, neuropathology, and clinical phenotype (ADI-R scores) in the brain tissues of individuals with ASD, we obtained RNA-seq data from GEO DataSets for TE profiling and identified host/target genes that may be influenced by TEs. Then, we examined the sequence characteristics and TF binding sites of differentially expressed TEs and predicted the biological functions of host and target genes. Finally, postmortem brain tissues obtained from the Harvard Brain Tissue Resource were used to investigate DNA methylation of Alu elements and neuropathology. Correlation analysis was conducted to investigate the relationship between the results.

### 4.1. Data Collection

To investigate TE expression profiling in the prefrontal cortex of ASD individuals, we obtained publicly available data from the NCBI Gene Expression Omnibus database (GEO data: https://www.ncbi.nlm.nih.gov/gds) accessed on 29 January 2020 using the following criteria: (i) ASD studies that used postmortem brain tissues from the prefrontal cortex of ASD and unaffected individuals; (ii) studies that used RNA sequencing and provided raw data; (iii) sample sizes greater than or equal to 40; and (iv) RNA-seq data of male individuals. A total of four studies were obtained [16,68,69,70]. RNA-seq raw data were obtained from the NCBI Sequence Read Archive repository (SRA: https://www.ncbi.nlm.nih.gov/sra) accessed on 29 August 2020.

### 4.2. RNA-Seq Data Reanalysis for Transposable Element Profiling in the Prefrontal Cortex of ASD Individuals

All RNA-seq data (FASTQ files) for ASD and unaffected individuals in the SRA repository were downloaded to the Galaxy program (https://usegalaxy.org) accessed on 12 August 2020, and analyses were performed using Galaxy program tools [71]. First, the FASTQ files were cleaned, and quality control was performed using the *fastp* package (fast all-in-one preprocessing for FASTQ files). For TE expression profiling, clean reads were mapped to the human reference genome (hg38) using RNA STAR aligner [72] with a parameter for multimapped reads according to Teissandier’s study [73]. To quantify TE transcripts, the mapped reads of individuals were counted using the Subread package FeatureCounts [74] with multimode (-M, -f). RepeatMasker annotation for hg38 was downloaded from the UCSC Table Browser [75]. To quantify protein-coding genes, the unique mode (-U) and the primary alignment mode of FeatureCounts were used for quantification with FeatureCounts built-in annotations for hg38. We excluded a few individuals from the differential expression analysis because their unique and multimapped ratios were markedly different.

Differential expression analyses of TEs and protein-coding genes were performed separately using the R package DESeq2 for ASD versus unaffected groups [76]. To normalize sequencing depth and RNA composition, read counts were normalized by the geometric mean method. Moreover, we normalized the data for other variable factors (i.e., library preparation and other unwanted technical effects) using the remove unwanted variation (RUV) tool [77]. Genes and TEs with a P and FDR of less than 0.01 were considered significant.

### 4.3. Identification of Genomic Loci of Differentially Expressed TEs Located Within/near Protein-Coding Genes

To determine whether TEs are associated with the expression level of protein-coding genes, we identified the genomic locations of each differentially expressed TE (DETE) located within or near a protein-coding gene region using the bedtools package [78]. First, the genomic loci of each TE were retrieved from RepeatMasker annotation for hg38 (http://repeatmasker.org) accessed on 1 November 2020, and NCBI RefSeq genes were downloaded from the UCSC Table Browser [79]. Then, the genomic loci of DETEs were intersected with the NCBI RefSeq genes (exonic regions) using the bedtools Intersect intervals function [78]. These processes were repeated with each intronic region and regions upstream of the TSS (50 kb, 10 kb, and 1 kb).

### 4.4. Cis- and Trans-Regulation of DETEs and the Expression Level of Nearby and Distant Genes

To determine whether dysregulation of TEs can affect host gene expression in cis- and trans-regulation, we performed Pearson’s correlation analyses between the normalized expression value (rlog transformation) of individual DETEs and their host genes. Correlations between expression of Alu elements and host genes with an adjusted P less than 0.05 (FDR = 0.05) were considered statistically significant. In this study, we also propose trans-regulation by Alu elements. We performed correlation analysis between all probabilities between 864 DETEs and 1493 DEGs, and an adjusted *p* < 0.05 (FDR = 0.05) was considered statistically significant.

### 4.5. Identification of Biological Functions, Pathways, and Autism Candidate Genes of Differentially Expressed Genes Associated with TEs

To predict the biological function and pathway of differentially expressed genes correlating significantly with TEs, the list of genes with log2-fold change and adjusted P were selected. Biological functions, pathways, and gene regulatory networks were predicted by IPA software (QIAGEN Inc., Hilden, Germany, https://www.qiagenbioinformatics.com/products/ingenuitypathway-analysis) accessed on 18 June 2021, and *p* < 0.05 was considered statistically significant. To identify autism candidate genes regulated by TEs in the prefrontal cortex of ASD individuals, genes were obtained from the SFARI database. The list of differentially expressed genes associated with TEs overlapped with 1003 autism candidate genes from the SFARI database [80] when using a Venn diagram drawing tool (Venny 2.1 software) [81], and gene set enrichment analysis was performed using the hypergeometric distribution calculator (http://keisan.casio.com/exec/system/1180573201) accessed on 22 February 2021.

### 4.6. Multiple Sequence Alignment Analysis and Transcription Factor Binding Site Prediction of Alu Elements

To determine the sequence characteristics of Alu elements differentially expressed in the postmortem brain tissues of ASD individuals, we extracted the sequence (FASTA) of each DE-Alu element from the human genome hg38 using the bedtools GetFastaBed function. The DE-Alu element sequences were analyzed using the multiple sequence alignment package (msa, R package) [82] by comparing the consensus sequence of DE-Alu elements with non-DE-Alu elements randomly chosen equally to the number of DETEs. The sequence logo and conserved scores were created using the ggseqlogo package [83].

For TF binding site prediction at the conserved region of DE-Alu elements, sequences were scanned using the matrix profile of a known TF binding site (*Homo sapiens*) in the JASPAR CORE database [84]. We selected all matrix profiles of *Homo sapiens* for scanning the conserved region identified by msa analysis and performed scanning with a relative score threshold >0.8.

### 4.7. DNA Extraction from Postmortem Brain Tissues

In this study, prefrontal cortices from ASD individuals (*n =* 7) and unaffected individuals (*n =* 6) were kindly provided by Dr. Valerie W. Hu (George Washington University), as originally obtained from the Harvard Brain Tissue Resource Center. First, approximately 100 mg of tissue was washed with PBS on ice three times. Then, lysis solution and 0.5 mm glass beads were added to the microcentrifuge tube. Homogenization was performed by using Disruptor Genie (Scientific Industries, Bohemia, NY, USA) at high speed at 4 °C for 2 min; this step was repeated if homogenization was incomplete. Acid phenol/chloroform was added to the homogenate, which was centrifuged at 10,000× *g* for 5 min. Genomic DNA was isolated using GENEzol reagent and precipitated by adding 100% ethanol to the interphase and the lower phenol–chloroform phase, followed by centrifugation at 2000× *g* for 5 min. The DNA pellet was resuspended in 0.1 M sodium citrate in 10% ethanol and incubated for 30 min before centrifuging and discarding the supernatant; this step was repeated twice. The DNA pellet was washed with 75% ethanol and air-dried for 5–10 min; the DNA pellet was resuspended and stored in 8 mM NaOH and 1 mM EDTA. The quality and quantity of purified DNA was measured using a NanoDrop One (Thermo Fisher Scientific, Inc., Rockingham County, NH, USA).

### 4.8. Combined Bisulfite Restriction Analysis (COBRA) for Alu Methylation Level and Patterns

The first step of the COBRA technique is bisulfite conversion, in which 1 µg of gDNA from human brain tissues was treated with sodium bisulfite using an EZ DNA Methylation-Gold™ Kit (Zymo, Irving, CA, USA) according to the manufacturer’s protocol. Then, the bisulfite-treated DNA was amplified by 35 cycles of PCR (Hot Start Taq DNA polymerase, QIAGEN, USA) using specific primers for the global AluSx family based on the AluSx consensus sequence. The PCR amplicons were digested with a restriction enzyme and incubated at 65 °C overnight, and the digested products were electrophoresed through an 8% nondenaturing polyacrylamide gel. The band intensity was measured by using GelAnalyzer 19.1 (http://www.gelanalyzer.com; accessed on 24 August 2020). The percentages of Alu methylation levels and patterns were calculated using the following formulas according to previous studies: percentage methylated loci (%^m^C) = 100 × (E + B)/(2A + E + B + C + D), percentage of the hypermethylated pattern (%^m^C^m^C) = 100 × F/(A + C + D + F), percentage of the partially methylated pattern (%^u^C^m^C) = 100 × C/(A + C + D + F), percentage of the partially methylated pattern (%^m^C^u^C) = 100 × D/(A + C + D + F), and percentage of the partially hypomethylated pattern (%^u^C^u^C) = 100 × A/(A + C + D + F) [20,21]. All samples were analyzed in duplicate, and the SK-N-FI cell line was used for interassay normalization.

For locus-specific DNA methylation of DE-Alu elements located within or near host genes, candidate DE-Alu elements were selected using the following criteria: (i) Alu elements must correlate with their host/distant gene expression. (ii) Alu elements must have CpG sites at the promoter region that can be detected by the COBRA method. (iii) Host/distant genes of Alu elements must be the candidate differentially expressed genes found in IPA and interactome analyses. (iv) Host/distant genes of Alu elements must be associated with the neuropathology of ASD postmortem brain tissues. The genomic locations of selected Alu elements were obtained from RepeatMasker annotation and used to retrieve the sequence of each Alu element from the UCSC table browser. To obtain the sequence for designing specific primers, we also selected the upstream region of Alu elements (100 bp upstream region plus + Alu sequence). The COBRA primer was designed using Bisulfite Primer Seeker (https://www.zymoresearch.com/pages/bisulfite-primer-seeker) accessed on 14 February 2022, with specific forward primers spanning the upstream region (host DEG sequence) and reverse primers within the Alu sequence. This strategy increases the specificity of the primer because Alu elements have a high copy number. We also verified the primers by performing gradient PCR and gel electrophoresis to select the optimal temperature. Finally, COBRA was performed and the %methylation level assessed as described in global methylation analysis.

### 4.9. Quantitative RT–PCR Analysis

Quantitative RT–PCR was performed to determine expression levels and the relative copy number of AluSx and to confirm the expression levels of selected differentially expressed genes from RNA-seq. Briefly, total RNA was isolated from postmortem brain tissues (ASD; *n =* 7, unaffected controls; *n =* 6) using a mirVana miRNA isolation kit (Ambion, Foster City, CA, USA) according to the manufacturer’s protocol. A total of 1 µg RNA was treated with DNaseI enzyme (Thermo Fisher Scientific, Waltham, MA, USA) before conversion to cDNA using a RevertAid RT reverse transcription kit (Thermo Fisher Scientific, USA). The qPCR assay was performed using RealMOD™ Green W^2^ 2× qPCR mix (iNtRON Biotechnology, Gyeonggi-do, Republic of Korea). Amplification was performed using a CFX Connect Real-Time PCR System (Bio-Rad, Hercules, CA, USA), consisting of an initial step at 95 °C for 15 min followed by 40 cycles of 45 s at 95 °C for denaturation and 30 s at 60 °C for annealing/extension. Product formation was confirmed by melting curve analysis (55 to 94 °C). The AluSx transcript-specific primers used were as follows: forward 5′-GTGGCTCACGCCTGTAATC-3′ and reverse 5′-GTAGAGACGGGGTTTCACCA-3′. The number of AluSx transcripts was normalized to 18S RNA and calculated using the 2^−ΔΔCt^ method.

For AluSx copy number quantification, we performed direct qPCR to amplify AluSx copies from genomic DNA as described in previous studies [85,86]. Briefly, 250 pg of genomic DNA was used directly for the qPCR assay using AccuPower^®^ 2× GreenStar™ qPCR MasterMix (Bioneer, Daejeon, Republic of Korea) according to the manufacturer’s instructions. For each sample, reactions were prepared in triplicate, in which the AluSx copy number was normalized by SATA elements and calculated using the 2^−ΔΔCt^ method.

### 4.10. Nissl Staining and Quantification of Neuron Number

To quantify neuronal cell number in the prefrontal cortex of ASD and unaffected individuals, paraffin-embedded tissues, also from the Harvard Brain Tissue Resource Center, were deparaffinized in xylene and stained with cresyl violet as follows: The tissue sections were deparaffinized in xylene for 10 min (2 steps) and then rehydrated in 100% ethanol solutions for 2 min (2 change steps) followed by decreasing concentrations of ethanol solution (1 step in 90% ethanol, 1 step in 80% ethanol, and 1 step in 70% ethanol; 1 min each step) and then placed in distilled water for 2 min. The deparaffinized tissue sections were stained with 0.5% cresyl violet (Sigma–Aldrich, St. Louis, MO, USA) for 2 min. Finally, the sections were rinsed in running tap water for 2 min followed by differentiation and dehydration in increasing concentrations of ethanol solution (1 step in 70% ethanol, 1 step in 80% ethanol, 1 step in 90% ethanol, and 2 steps in 100% ethanol; 5 min each step). The sections were captured by light microscopy (DM1000; Leica Microsystems, Wetzlar, Germany). We quantified neuronal cell numbers in three selected regions of each prefrontal cortex that covered the complete thickness of the cortical gray matter. We identified neurons based on their unique morphological characteristics by Nissl staining [87].

### 4.11. Statistical Analysis

Statistical analyses were performed using IBM SPSS version 22. For comparison of means, a two-tailed Student’s *t* test was used. A *p* value less than 0.05 was considered statistically significant. Pearson’s correlation analysis was employed for cis- and trans-regulation, DNA methylation, gene expression, neuron density, and ADI-R scores. For multiple test comparisons, the Benjamini–Hochberg correction method was applied at an FDR < 0.05. The statistical threshold for DETEs and DEGs was FDR = 0.01.

## 5. Conclusions

TE expression profiling revealed the Alu family to be dysregulated in the prefrontal cortex of ASD individuals. Moreover, a dysregulation of individual Alu elements correlated with host/nearby gene expression levels in cis- and trans-regulation. The DEGs associated with Alu elements are related to ASD and the neuropathology of ASD, and some of these genes are autism candidates in the SFARI database. In addition, COBRA analysis showed a change in the percentage of methylated patterns of two CpG sites in the Alu promoter in a subpopulation of individuals with ASD. Our findings provide a better understanding and highlight the role of Alu elements in gene dysregulation in the prefrontal cortex of ASD individuals. Further investigation of the relationship between Alu elements, gene regulation, and neuropathology in larger ASD sample sizes is warranted.

## Figures and Tables

**Table 1:** Number of DETE loci used for correlation with their hosts/nearby DEGs in the prefrontal cortex of ASD individuals. DETE loci were classified based on genomic features and considered significant by a Benjamini–Hochberg (BH)-adjusted *p* < 0.05.

Genomic Feature	DETEs Position	No. of DETEs Located in DEGs	No. of Significantly Correlating Loci		
			All	Control	ASD
All transposable elements					
All features	18,045	1587	1109	390	534
50 kb upstream region	8199	507	293	63	119
10 kb upstream region	1893	120	82	19	26
1 kb upstream region	236	9	7	1	3
Intronic region	7232	744	522	136	195
Exonic region	485	207	205	171	191
Alu elements					
All features	-	659	417	86	161
50 kb upstream region	-	328	181	33	67
Intronic region	-	291	197	27	58
Exonic region	-	40	39	26	36

**Table 2:** Correlation analyses for trans-regulation in prefrontal cortex tissues. Statistical analysis: Pearson’s correlation with multiple hypothesis correction (FDR = 0.05).

Trans-Regulation	DETEs-DEGs	Correlation Probability	ASD		Control	
			Significant Probability	DETE Elements	Significant Probability	DETE Elements
Alu elements	No. correlation loci	491,198	40,599	316	7141	196
	No. DEGs	1493	1465	-	1239	-

**Table 3:** List of unique ASD-associated TFs binding at the DE-AluS consensus sequence predicted by JASPAR CORE database. “+” represents the sense strand and “–“ represents the antisense strand.

Matrix ID	Symbol	Relative Score	Sequence ID	Start	End	Strand	Predicted Sequence
MA1535.1	NR2C1	0.98463	DEAluS	8	16	+	TGAGGTCAG
MA1535.1	NR2C1	0.81979	DEAluS	16	24	+	GGAGTTCAA
MA0018.2	CREB1	0.9342	DEAluS	8	15	+	TGAGGTCA
MA0018.2	CREB1	0.86424	DEAluS	8	15	−	TGACCTCA
MA0018.4	CREB1	0.85236	DEAluS	5	17	+	ACTTGAGGTCAGG
MA0018.3	CREB1	0.84877	DEAluS	6	17	+	CTTGAGGTCAGG
MA0018.3	CREB1	0.84877	DEAluS	6	17	−	CCTGACCTCAAG
MA0684.2	RUNX3	0.92281	DEAluS	5	16	−	CTGACCTCAAGT
MA0684.1	RUNX3	0.80775	DEAluS	6	15	−	TGACCTCAAG
MA1534.1	NR1I3	0.92153	DEAluS	16	24	−	TTGAACTCC
MA1534.1	NR1I3	0.85571	DEAluS	8	16	−	CTGACCTCA
MA0017.2	NR2F1	0.91535	DEAluS	7	19	+	TTGAGGTCAGGAG
MA0693.2	VDR	0.90919	DEAluS	16	23	+	GGAGTTCA
MA0693.2	VDR	0.89944	DEAluS	8	15	+	TGAGGTCA
MA0160.1	NR4A2	0.90185	DEAluS	9	16	+	GAGGTCAG
MA0160.1	NR4A2	0.82039	DEAluS	23	30	+	AAGACCAG
MA0160.2	NR4A2	0.80399	DEAluS	6	15	+	CTTGAGGTCA
MA0672.1	NKX2-3	0.89714	DEAluS	2	11	+	ATCACTTGAG
MA1111.1	NR2F2	0.87947	DEAluS	7	17	+	TTGAGGTCAGG
MA1587.1	ZNF135	0.87829	DEAluS	13	26	−	TCTTGAACTCCTGA
MA1531.1	NR1D1	0.87418	DEAluS	10	24	+	AGGTCAGGAGTTCAA
MA0909.1	HOXD13	0.86899	DEAluS	58	67	+	CTACTAAAAA
MA0093.2	USF1	0.8659	DEAluS	1	11	−	CTCAAGTGATT
MA0093.1	USF1	0.82827	DEAluS	3	9	−	CAAGTGA
MA0093.1	USF1	0.8266	DEAluS	4	10	+	CACTTGA
MA0093.2	USF1	0.82641	DEAluS	11	21	−	AACTCCTGACC
MA1532.1	NR1D2	0.86489	DEAluS	10	24	+	AGGTCAGGAGTTCAA
MA1532.2	NR1D2	0.84991	DEAluS	9	23	+	GAGGTCAGGAGTTCA
MA0871.1	TFEC	0.86383	DEAluS	2	11	+	ATCACTTGAG
MA0871.1	TFEC	0.85006	DEAluS	2	11	−	CTCAAGTGAT
MA0871.2	TFEC	0.8011	DEAluS	1	11	−	CTCAAGTGATT
MA0154.2	EBF1	0.8628	DEAluS	3	13	−	ACCTCAAGTGA
MA0154.2	EBF1	0.84908	DEAluS	2	12	+	ATCACTTGAGG
MA0154.1	EBF1	0.84704	DEAluS	3	12	−	CCTCAAGTGA
MA1632.1	ATF2	0.86072	DEAluS	5	17	+	ACTTGAGGTCAGG
MA0901.1	HOXB13	0.85918	DEAluS	58	67	+	CTACTAAAAA
MA0158.1	HOXA5	0.85801	DEAluS	4	11	−	CTCAAGTG
MA0158.1	HOXA5	0.82169	DEAluS	14	21	+	CAGGAGTT
MA0650.1	HOXA13	0.85681	DEAluS	58	67	+	CTACTAAAAA
MA1649.1	ZBTB12	0.85649	DEAluS	17	27	−	GTCTTGAACTC
MA0058.1	MAX	0.85576	DEAluS	1	10	+	AATCACTTGA
MA0831.2	TFE3	0.85542	DEAluS	2	9	−	CAAGTGAT
MA0831.1	TFE3	0.83464	DEAluS	12	21	−	AACTCCTGAC
MA0831.2	TFE3	0.83003	DEAluS	12	19	−	CTCCTGAC
MA0831.3	TFE3	0.81998	DEAluS	2	11	−	CTCAAGTGAT
MA0831.3	TFE3	0.81998	DEAluS	2	11	+	ATCACTTGAG
MA0511.2	RUNX2	0.85286	DEAluS	7	15	−	TGACCTCAA
MA0899.1	HOXA10	0.85	DEAluS	57	67	+	TCTACTAAAAA
MA0071.1	RORA	0.84916	DEAluS	6	15	+	CTTGAGGTCA
MA0828.1	SREBF2	0.84851	DEAluS	2	11	−	CTCAAGTGAT
MA0828.1	SREBF2	0.83605	DEAluS	2	11	+	ATCACTTGAG
MA0828.1	SREBF2	0.81121	DEAluS	12	21	−	AACTCCTGAC
MA0036.1	GATA2	0.84608	DEAluS	1	5	−	TGATT
MA0464.2	BHLHE40	0.84575	DEAluS	2	11	−	CTCAAGTGAT
MA0464.2	BHLHE40	0.82176	DEAluS	2	11	+	ATCACTTGAG
MA0745.1	SNAI2	0.84549	DEAluS	3	11	−	CTCAAGTGA
MA0745.1	SNAI2	0.8117	DEAluS	24	32	−	GGCTGGTCT
MA0636.1	BHLHE41	0.84511	DEAluS	2	11	+	ATCACTTGAG
MA0636.1	BHLHE41	0.83972	DEAluS	2	11	−	CTCAAGTGAT
MA0729.1	RARA	0.83842	DEAluS	9	26	+	GAGGTCAGGAGTTCAAGA
MA1112.1	NR4A1	0.83437	DEAluS	7	16	+	TTGAGGTCAG
MA1902.1	NFkb	0.83413	DEAluS	37	58	+	CAACATGGTGAAACCCCGTCTC
MA0913.2	HOXD9	0.8321	DEAluS	58	67	+	CTACTAAAAA
MA0122.2	NKX3-2	0.83136	DEAluS	2	10	+	ATCACTTGA
MA1655.1	ZNF341	0.8311	DEAluS	23	34	+	AAGACCAGCCTG
MA0833.2	ATF4	0.83022	DEAluS	39	52	+	ACATGGTGAAACCC
MA1656.1	ZNF449	0.82761	DEAluS	21	34	+	TCAAGACCAGCCTG
MA1963.1	SATB1	0.82693	DEAluS	55	67	+	TCTCTACTAAAAA
MA1464.1	ARNT2	0.82595	DEAluS	2	11	+	ATCACTTGAG
MA1558.1	SNAI1	0.82506	DEAluS	2	11	−	CTCAAGTGAT
MA1558.1	SNAI1	0.80459	DEAluS	12	21	+	GTCAGGAGTT
MA0100.3	MYB	0.8245	DEAluS	2	11	−	CTCAAGTGAT
MA1505.1	HOXC8	0.82307	DEAluS	3	10	−	TCAAGTGA
MA0664.1	MLXIPL	0.8215	DEAluS	2	11	−	CTCAAGTGAT
MA0664.1	MLXIPL	0.80617	DEAluS	2	11	+	ATCACTTGAG
MA0002.1	RUNX1	0.82024	DEAluS	5	15	+	ACTTGAGGTCA
MA0842.1	NRL	0.81706	DEAluS	12	22	−	GAACTCCTGAC
MA0663.1	MLX	0.81579	DEAluS	2	11	−	CTCAAGTGAT
MA0525.1	TP63	0.81575	DEAluS	19	38	+	GTTCAAGACCAGCCTGGCCA
MA0007.2	AR	0.81533	DEAluS	23	37	+	AAGACCAGCCTGGCC
MA0063.2	NKX2-5	0.81522	DEAluS	2	12	+	ATCACTTGAGG
MA0063.2	NKX2-5	0.80188	DEAluS	57	67	+	TCTACTAAAAA
MA1563.1	SOX18	0.81158	DEAluS	39	46	−	CACCATGT
MA1496.1	HOXA4	0.81143	DEAluS	12	19	+	GTCAGGAG
MA1496.1	HOXA4	0.8107	DEAluS	41	48	+	ATGGTGAA
MA1151.1	RORC	0.81043	DEAluS	4	15	+	CACTTGAGGTCA
MA0692.1	TFEB	0.80964	DEAluS	2	11	−	CTCAAGTGAT
MA0692.1	TFEB	0.80394	DEAluS	12	21	−	AACTCCTGAC
MA0692.1	TFEB	0.80321	DEAluS	2	11	+	ATCACTTGAG
MA1570.1	TFAP4	0.80899	DEAluS	36	45	−	ACCATGTTGG
MA1570.1	TFAP4	0.80622	DEAluS	36	45	+	CCAACATGGT
MA0763.1	ETV3	0.80785	DEAluS	4	13	−	ACCTCAAGTG
MA1498.1	HOXA7	0.80731	DEAluS	20	27	−	GTCTTGAA
MA1540.1	NR5A1	0.80706	DEAluS	6	16	+	CTTGAGGTCAG
MA1540.1	NR5A1	0.8025	DEAluS	20	30	+	TTCAAGACCAG
MA0492.1	JUND	0.80596	DEAluS	3	17	+	TCACTTGAGGTCAGG
MA0523.1	TCF7L2	0.805	DEAluS	16	29	+	GGAGTTCAAGACCA
MA0819.2	CLOCK	0.8047	DEAluS	37	46	−	CACCATGTTG
MA0522.2	TCF3	0.8032	DEAluS	12	21	−	AACTCCTGAC
MA0820.1	FIGLA	0.80267	DEAluS	12	21	−	AACTCCTGAC
MA0488.1	JUN	0.80233	DEAluS	4	16	+	CACTTGAGGTCAG
MA1549.1	POU6F1	0.80132	DEAluS	12	21	+	GTCAGGAGTT
MA1541.1	NR6A1	0.8013	DEAluS	14	30	+	CAGGAGTTCAAGACCAG
MA0827.1	OLIG3	0.80016	DEAluS	36	45	+	CCAACATGGT

**Table 4:** COBRA-derived percentages of AluSx methylation levels and patterns in the prefrontal cortex of ASD individuals and sex- and age-matched unaffected controls.

AluSx Methylation	%^m^C	%^m^C^m^C	%^u^C^m^C	%^m^C^u^C	%^u^C^u^C
ASD	38.837	26.496	21.128	19.748	32.629
Ctrl	39.304	27.634	20.843	19.254	32.270
*p* value (Effect size, Cohen’s d)	0.201 (−0.756)	0.104 (−0.985)	0.037 (1.324)	0.133 (0.904)	0.348 (0.546)
NV-ASD	38.986	26.873	21.105	19.502	32.519
Ctrl	39.165	27.282	20.951	19.396	32.370
*p* value (Effect size, Cohen’s d)	0.726 (0.042)	0.661 (−0.185)	0.373 (0.807)	0.809 (0.270)	0.789 (−0.209)
V-ASD	38.638	25.992	21.158	20.075	32.775
Ctrl	39.797	28.642	20.696	18.849	31.812
*p* value (Effect size, Cohen’s d)	0.019 (−3.131)	0.004 (−4.826)	0.023 (2.925)	0.031 (2.666)	0.086 (1.853)

**Table 5:** Details of selected DEGs and DETEs from correlation analysis (cis-regulation) using RNA-seq data from the prefrontal cortex of ASD and unaffected individuals.

DEGs	log_2_(FC) DEGs	*p*-adj DEGs	DETEs	log_2_(FC) DETEs	*p*-adj DETEs	Type	R_All (BH-*p*)	R_Ctrl (BH-*p*)	R_ASD (BH-*p*)
*ATP8A2*	−0.64	2.82 × 10^−3^	AluSq2	1.32	1.00 × 10^−4^	Intron	−0.83 (3.27 × 10^−10^)	−0.70 (1.60 × 10^−3^)	−0.81 (5.37 × 10^−05^)
*ATP8A2*	−0.64	2.82 × 10^−3^	AluSg7	1.03	7.11 × 10^−3^	Intron	−0.57 (3.83 × 10^−4^)	0.11 (7.28 × 10^−1^)	−0.62 (8.92 × 10^−3^)
*ATP8A2*	−0.64	2.82 × 10^−3^	AluJb	1.24	1.55 × 10^−3^	Intron	−0.48 (4.57 × 10^−3^)	−0.22 (4.73 × 10^−1^)	−0.32 (2.60 × 10^−1^)
*HMOX1*	1.76	3.07 × 10^−6^	AluSg7	1.31	3.69 × 10^−3^	Intron	0.77 (3.53 × 10^−8^)	−0.11 (7.25 × 10^−1^)	0.86 (5.23 × 10^−6^)
*PARP9*	1.03	1.26 × 10^−3^	AluY	1.22	6.30 × 10^−3^	Intron	0.70 (1.96 × 10^−6^)	0.40 (1.34 × 10^−1^)	0.69 (2.26 × 10^−3^)
*GRIN2A*	−0.87	6.51 × 10^−4^	AluSz6	−1.04	1.65 × 10^−3^	Intron	0.79 (1.32 × 10^−8^)	0.60 (1.24 × 10^−2^)	0.71 (1.44 × 10^−3^)
*GRIN2A*	−0.87	6.51 × 10^−4^	AluSp	1.34	5.76 × 10^−4^	Intron	−0.67 (1.15 × 10^−05^)	−0.20 (5.00 × 10^−1^)	−0.62 (8.64 × 10^−3^)
*GRIN2B*	−0.76	3.73 × 10^−3^	AluSg4	1.81	4.59 × 10^−4^	Intron + 50 kb	−0.50 (2.67 × 10^−3^)	−0.11 (7.33 × 10^−1^)	−0.62 (8.84 × 10^−3^)
*GRIN2B*	−0.76	3.73 × 10^−3^	AluSx1	1.32	5.91 × 10^−3^	Intron + 50 kb	−0.45 (8.49 × 10^−3^)	−0.26 (3.67 × 10^−1^)	−0.34 (2.27 × 10^−1^)
*DOCK1*	0.77	7.08 × 10^−4^	AluY	1.08	5.41 × 10^−3^	Intron	0.72 (8.93 × 10^−7^)	0.51 (4.50 × 10^−2^)	0.71 (1.50 × 10^−3^)
*RNF135*	0.76	8.96 × 10^−4^	AluJr	1.17	3.68 × 10^−3^	exon	0.79 (1.08 × 10^−8^)	0.66 (4.08 × 10^−3^)	0.78 (1.92 × 10^−4^)
*RNF135*	0.76	8.96 × 10^−4^	AluJb	1.41	2.37 × 10^−3^	50 kb	0.78 (2.48 × 10^−8^)	0.62 (8.84 × 10^−3^)	0.79 (1.19 × 10^−4^)
*RNF135*	0.76	8.96 × 10^−4^	AluYk3	1.31	4.24 × 10^−3^	50 kb	0.71 (1.71 × 10^−6^)	0.44 (9.05 × 10^−2^)	0.73 (7.43 × 10^−4^)
*KCNQ5*	−0.63	4.90 × 10^−3^	AluSc	1.48	5.65 × 10^−3^	Intron + 50 kb	−0.59 (2.07 × 10^−4^)	0.21 (4.88 × 10^−1^)	−0.66 (4.15 × 10^−3^)
*KCNQ5*	−0.63	4.90 × 10^−3^	AluSz	1.15	3.13 × 10^−3^	Intron + 50 kb	−0.41 (2.01 × 10^−2^)	−0.08 (8.13 × 10^−1^)	−0.31 (2.70 × 10^−1^)

## Data Availability

The datasets used as the main analysis in this study were obtained from GEO DataSets (GSE51264 and GSE59288).

## Supplementary Materials

The following supporting information can be downloaded at https://www.mdpi.com/article/10.3390/ijms24087518/s1.
