# Supplementary material for: Epigenetic Gene-Regulatory Loci in Alu Elements Associated with Autism Susceptibility in the Prefrontal Cortex of ASD
Source: Int J Mol Sci. 2023 Apr 19;24(8):7518. doi: 10.3390/ijms24087518 (PMC10139202; doi:10.3390/ijms24087518)
Supplement: Supplementary file 1 [file ijms-24-07518-s001.zip › Figure S2.pdf]

Figure S2a Hypergeometric distribution analysis. (a) Overlapping between DEGs correlated with DETEs in ASD individuals and SFARI genes. (b) Overlapping between all DEGs in ASD individuals and SFARI genes.

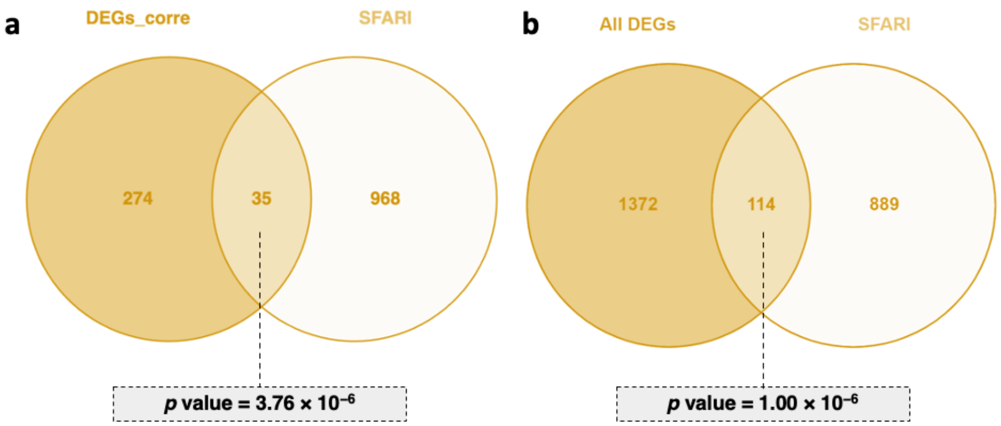

Figure S2b. List of overlapping genes between correlated DEGs classified by Alu location and autism candidate genes from the SFARI database.

Overlapping	#DEGs	List of overlapping DEGs
SFARI versus DEGs-Alu within intron	24	*BAZ2B,BCAS1,CNTNAP2,CSMD1,DOCK1,GABBR2,GABRG3,GRIN2A,GRIN2B,KANK1,MYH10,MYO9B,NBEA,NRXN3,PCDH19,PCDHA1,PCDHA3,PCDHA4,PCDHA5,PCDHA6,PTPRT,RNF135,UNC5D,XPO1*
SFARI versus DEGs-Alu within 50kb	14	*EGR3,GATM,GRIN2B,MEF2C,MYO9B,NFIX,PCDHA11,PCDHA12,RNF135,STXBP1,VAMP2,UNC5D,XPO1,YWHAG*
SFARI versus DEGs-Alu within exon	3	*DAPP1,KCNB1,RNF135*
