# Supplementary material for: Epigenetic Gene-Regulatory Loci in Alu Elements Associated with Autism Susceptibility in the Prefrontal Cortex of ASD
Source: Int J Mol Sci. 2023 Apr 19;24(8):7518. doi: 10.3390/ijms24087518 (PMC10139202; doi:10.3390/ijms24087518)

Figure S3 Consensus sequences and conserve scores of DE-AluY and nonDE-AluY.
This figure showed the consensus sequence of AluY subfamily at the left arm of elements (containing the promoter region).

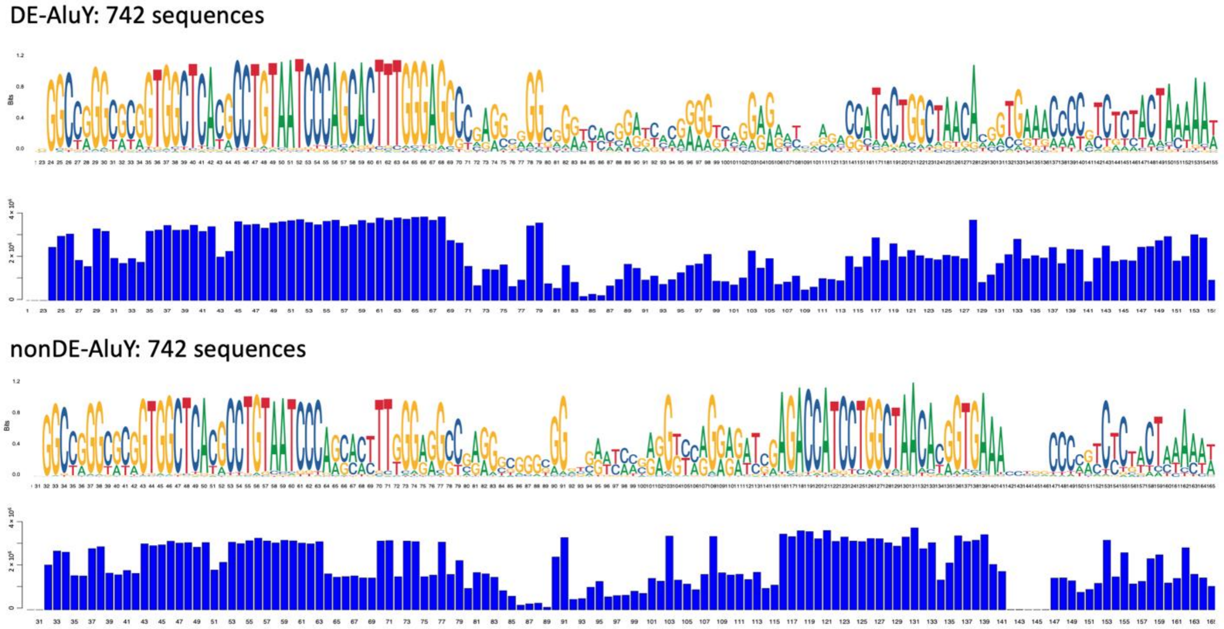

Supplement: Supplementary file 1 [file ijms-24-07518-s001.zip › Figure S3.pdf]
