# Supplementary material for: Epigenetic Gene-Regulatory Loci in Alu Elements Associated with Autism Susceptibility in the Prefrontal Cortex of ASD
Source: Int J Mol Sci. 2023 Apr 19;24(8):7518. doi: 10.3390/ijms24087518 (PMC10139202; doi:10.3390/ijms24087518)
Supplement: Supplementary file 1 [file ijms-24-07518-s001.zip › Table S1.pdf]

Table S1. Details of the transcriptome data using post-mortem brain tissues of ASD and unaffected individuals obtained from GEO DataSets.

Data type	GSE number	Author	Sample type	Sample size	Titles	Notes
RNA-seq data	GSE59288	Liu et al. (2016)	Prefrontal cortex	ASD=34, Ctrl=38	Disruption of an Evolutionarily Novel Synaptic Expression Pattern in Autism	RNA-seq RAW data
	GSE102741	Wright et al. (2017)	Prefrontal cortex	ASD=13, Ctrl=39	Altered expression of histamine signaling genes in autism spectrum disorder.	RNA-seq RAW data
	GSE64018	Parikshak et al. (2014)	Prefrontal cortex and cerebellum	ASD=12, Ctrl=12	Genome-wide changes in lncRNA, splicing, and regional gene expression patterns in autism	no available RAW data
	GSE30573	Voineagu et al. (2011)	Prefrontal cortex	ASD=3, Ctrl=3	Transcriptomic analysis of autistic brain reveals convergent molecular pathology	RNA-seq RAW data
