# Supplementary material for: Epigenetic Gene-Regulatory Loci in Alu Elements Associated with Autism Susceptibility in the Prefrontal Cortex of ASD
Source: Int J Mol Sci. 2023 Apr 19;24(8):7518. doi: 10.3390/ijms24087518 (PMC10139202; doi:10.3390/ijms24087518)
Supplement: Supplementary file 1 [file ijms-24-07518-s001.zip › Table S5.pdf]

Table S5. Diseases and functions of the DEGs correlated with DETEs and DE-Alu in the prefrontal cortex of ASD predicted by IPA.

Diseases or Functions Annotation	DETEs	DE-Alu	
	p value (#genes)	*p* value (#genes)	DEGs lists
Pervasive developmental disorder	1.04×10^{-7} (21)	6.97×10^{-5} (9)	*CNTNAP2,GNG5,GRIN2A,GRIN2B,IL18,RNF135, TSPO,USP3,VAMP2*
Autism spectrum disorder or intellectual disability	4.33×10^{-5} (37)	1.19×10^{-3} (16)	*ATP8A2,CNTNAP2,GNG5,GRIN2A,GRIN2B,IL18, KCNB1,KCNQ5,NBEA,RNF135,SCN3B,STXBP1,TRPC5,TSPO,USP3,VAMP2*
Nonspecific mental retardation	8.84×10^{-5} (19)	3.81×10^{-3} (8)	*CNTNAP2,GRIN2A,GRIN2B,KCNB1,KCNQ5,NBEA,STXBP1,TRPC5*
Seizures	5.29×10^{-8} (25)	1.80×10^{-3} (9)	*CNTNAP2,GRIN2A,GRIN2B,HMOX1,KCNB1,NBEA,SCN3B,STXBP1,TSPO*
Movement Disorders	4.94×10^{-7} (45)	1.52×10^{-3} (17)	*ATP8A2,CDH18,CFLAR,GRIN2A,GRIN2B,HMOX1,ID3,KCNB1,NBEA,NDRG3,NEDD4,RPH3A,SCN3B,SLIT2,SPP1,TSPO,VAMP2*
Epilepsy	6.94×10^{-6} (22)	8.46×10^{-3} (8)	*CNTNAP2,GRIN2A,GRIN2B,HMOX1,KCNB1,SCN3B,STXBP1,TSPO*
Disorder of basal ganglia	9.62×10^{-6} (35)	2.12×10^{-3} (14)	*CFLAR,GRIN2A,GRIN2B,HMOX1,ID3,NBEA,NDRG3,NEDD4,RPH3A,SCN3B,SLIT2,SPP1,TSPO,VAMP2*
Multiple Sclerosis	1.00×10^{-5} (17)	6.05×10^{-4} (8)	*CFLAR,GRIN2A,GRIN2B,HAVCR2,KCNB1,MERTK,SPP1,TSPO*
Progressive neurological disorder	1.70×10^{-5} (49)	6.02×10^{-3} (19)	*APOC1,BRCA1,CFLAR,GRIN2A,GRIN2B,HAVCR2,HMOX1,KCNB1,MERTK,mir-612,MTHFD2,NEDD4,SCN3B,SLIT2,SPP1,STXBP1,TSPO,TYROBP,VAMP2*
Huntington Disease	3.29×10^{-5} (26)	4.75×10^{-4} (12)	*CFLAR,GRIN2A,GRIN2B,HMOX1,ID3,NBEA,NDRG3,RPH3A,SCN3B,SLIT2,TSPO,VAMP2*
Apoptosis	7.86×10^{-6} (70)	5.75×10^{-4} (30)	*APOC1,BAZ1A,BNIP2,BRCA1,CARD8,CFLAR,CHEK2,GPLD1,GRIN2B,HMOX1,ID3,IFIT3,IKZF1,IL18,MDM4,MERTK,MOAP1,NEDD4,PARP14,PLIN2,PTK6,S100A11,SCN3B,SLIT2,SPAG5,SPP1,TCF12,TGFB3,XAF1,XPO1*
