# Supplementary material for: Epigenetic Gene-Regulatory Loci in Alu Elements Associated with Autism Susceptibility in the Prefrontal Cortex of ASD
Source: Int J Mol Sci. 2023 Apr 19;24(8):7518. doi: 10.3390/ijms24087518 (PMC10139202; doi:10.3390/ijms24087518)
Supplement: Supplementary file 1 [file ijms-24-07518-s001.zip › Table S6.pdf]

Table S6. Canonical pathways of the DEGs correlated with DETEs and DE-Alu in the prefrontal cortex of ASD predicted by IPA.

Ingenuity Canonical Pathways	DETEs	DE-Alu	
	p value	*p* value	Molecules
Synaptogenesis Signaling Pathway	7.08×10^{-5}	8.91×10^{-5}	*ARPC1B,CDH12,CDH18,CNTNAP2,GRIN2A,GRIN2B,STXBP1,SYT13,VAMP2*
Calcium Signaling	7.76×10^{-5}	3.55×10^{-2}	*CAMK1D,GRIN2A,GRIN2B,TRPC5*
Fcγ Receptor-mediated Phagocytosis in Macrophages and Monocytes	8.13×10^{-5}	2.29×10^{-3}	*ARPC1B,DOCK1,GPLD1,HMOX1*
Neuroinflammation Signaling Pathway	2.34×10^{-4}	2.29×10^{-3}	*CFLAR,GRIN2A,GRIN2B,HMOX1,IL18,TGFB3,TYROBP*
Ephrin Receptor Signaling	3.31×10^{-3}	3.09×10^{-2}	*ARPC1B,GNG5,GRIN2A,GRIN2B*
Reelin Signaling in Neurons	1.17×10^{-2}	3.80×10^{-2}	*ARPC1B,GRIN2A,GRIN2B*
Glutamate Receptor Signaling	1.66×10^{-2}	6.76×10^{-3}	*GNG5,GRIN2A,GRIN2B*
Axonal Guidance Signaling	1.66×10^{-2}	1.63×10^{-1}	*ARPC1B,DOCK1,GNG5,SLIT2,UNC5D*
Choline Biosynthesis III	1.82×10^{-2}	3.02×10^{-3}	*GPLD1,HMOX1*
nNOS Signaling in Neurons	3.16×10^{-2}	3.02×10^{-2}	*GRIN2A,GRIN2B*
Cell Cycle: G2/M DNA Damage Checkpoint Regulation	3.98×10^{-2}	3.24×10^{-3}	*BRCA1,CHEK2,MDM4*
