# Supplementary material for: Epigenetic Gene-Regulatory Loci in Alu Elements Associated with Autism Susceptibility in the Prefrontal Cortex of ASD
Source: Int J Mol Sci. 2023 Apr 19;24(8):7518. doi: 10.3390/ijms24087518 (PMC10139202; doi:10.3390/ijms24087518)
Supplement: Supplementary file 1 [file ijms-24-07518-s001.zip › Table S7.pdf]

Table S7. List of the unique ASD-associated transcription factors which bonded at the DE-AluS consensus sequence predicted by the JASPAR CORE and overlapped with the SFARI database.

Matrix ID	Symbol	Score	Relative score	Sequence ID	Start	End	Strand	Predicted sequence
MA0017.2	NR2F1	12.2558	0.91535	DEAluS	7	19	+	TTGAGGTCAGGAG
MA0693.2	VDR	8.61672	0.90919	DEAluS	16	23	+	GGAGTTCA
MA0160.1	NR4A2	8.84519	0.90185	DEAluS	9	16	+	GAGGTCAG
MA0693.2	VDR	8.27478	0.89944	DEAluS	8	15	+	TGAGGTCA
MA1531.1	NR1D1	10.4393	0.87418	DEAluS	10	24	+	AGGTCAGGAGTTCAA
MA0071.1	RORA	8.90167	0.84916	DEAluS	6	15	+	CTTGAGGTCA
MA1963.1	SATB1	6.78882	0.82693	DEAluS	55	67	+	TCTCTACTAAAAA
MA1464.1	ARNT2	4.32459	0.82595	DEAluS	2	11	+	ATCACTTGAG
MA0160.1	NR4A2	6.52681	0.82039	DEAluS	23	30	+	AAGACCAG
MA0007.2	AR	5.42626	0.81533	DEAluS	23	37	+	AAGACCAGCCTGGCC
MA0523.1	TCF7L2	4.52663	0.805	DEAluS	16	29	+	GGAGTTCAAGACCA
MA0160.2	NR4A2	6.03833	0.80399	DEAluS	6	15	+	CTTGAGGTCA
