# Supplementary material for: Epigenetic Gene-Regulatory Loci in Alu Elements Associated with Autism Susceptibility in the Prefrontal Cortex of ASD
Source: Int J Mol Sci. 2023 Apr 19;24(8):7518. doi: 10.3390/ijms24087518 (PMC10139202; doi:10.3390/ijms24087518)
Supplement: Supplementary file 1 [file ijms-24-07518-s001.zip › Table S8.pdf]

Table S8. Locus-specific methylation of Alu elements located within/nearby host DEGs in the prefrontal cortex tissues of ASD and unaffected individuals.

DETEs	Host DEGs	Group	% CpG no1	% CpG no2	% CpG no3	% ^{m}C	% $^{m}C^{m}C$	% $^{m}C^{u}C$	% $^{u}C^{m}C$	% $^{u}C^{u}C$
AluYk3	*RNF135*	CTRL	90.96	90.17	70.73	95.70	60.41	37.08	1.23	1.28
		ASD	89.68	88.88	70.29	94.83	60.26	36.75	1.27	1.72
		p value	0.525	0.661	0.847	0.514	0.943	0.860	0.878	0.391
		CTRL	92.07	91.87	69.31	96.21	61.81	35.94	1.03	1.22
		NV-ASD	89.06	85.08	71.13	93.30	58.46	37.61	1.88	2.05
		p value	0.341	**0.033**	0.490	0.072	0.150	0.418	**0.013**	0.219
		CTRL	91.54	89.93	71.10	95.87	60.30	37.24	1.36	1.10
		V-ASD	90.30	93.46	68.33	96.63	62.90	35.00	0.87	1.24
		p value	0.739	0.380	0.478	0.653	0.455	0.501	0.229	0.819
AluSz6	*GRIN2A*	CTRL	83.04	58.87	-	77.33	52.33	39.71	4.73	3.22
		ASD	83.30	69.03	-	83.86	65.20	29.31	2.96	2.53
		p value	0.946	0.122	-	0.059	0.051	0.094	0.189	0.519
		CTRL	83.52	59.63	-	78.40	53.11	39.19	4.75	2.96
		NV-ASD	84.86	64.15	-	81.35	59.69	34.49	2.90	2.92
		p value	0.816	0.603	-	0.528	0.434	0.573	0.384	0.984
		CTRL	80.60	60.53	-	77.80	52.65	38.45	6.09	2.82
		V-ASD	81.74	73.91	-	86.37	70.71	24.14	3.02	2.13
		p value	0.865	0.246	-	0.152	0.116	0.172	0.196	0.662
AluSg7	*HMOX1*	CTRL	96.72	-	-	-	-	-	-	-
		ASD	96.93	-	-	-	-	-	-	-
		p value	0.724	-	-	-	-	-	-	-
		CTRL	96.69	-	-	-	-	-	-	-
		NV-ASD	97.11	-	-	-	-	-	-	-
		p value	0.651	-	-	-	-	-	-	-
		CTRL	96.21	-	-	-	-	-	-	-
		V-ASD	96.68	-	-	-	-	-	-	-
		p value	0.596	-	-	-	-	-	-	-
